# B-Cell Responses to Human Bocaviruses 1–4: New Insights from a Childhood Follow-Up Study

**DOI:** 10.1371/journal.pone.0139096

**Published:** 2015-09-29

**Authors:** Kalle Kantola, Lea Hedman, Laura Tanner, Ville Simell, Marjaana Mäkinen, Juulia Partanen, Mohammadreza Sadeghi, Riitta Veijola, Mikael Knip, Jorma Ilonen, Heikki Hyöty, Jorma Toppari, Olli Simell, Klaus Hedman, Maria Söderlund-Venermo

**Affiliations:** 1 University of Helsinki, Department of Virology, Helsinki, Finland; 2 Helsinki University Hospital Laboratory Services, Helsinki, Finland; 3 Turku University Hospital, Department of Pediatrics, Turku, Finland; 4 Medicity, University of Turku, Turku, Finland; 5 University of Oulu, Department of Pediatrics, Oulu, Finland; 6 University of Helsinki and Helsinki University Hospital, Children's Hospital and Research Programs Unit, Diabetes and Obesity, Helsinki, Finland; 7 Folkhälsan Research Center, Helsinki, Finland; 8 Tampere University Hospital, Tampere Center for Child Health Research, Tampere, Finland; 9 University of Eastern Finland, Department of Clinical Microbiology, Kuopio, Finland; 10 University of Turku, Immunogenetics Laboratory, Turku, Finland; 11 University of Tampere, Department of Virology, Tampere, Finland; Kliniken der Stadt Köln gGmbH, GERMANY

## Abstract

Human bocaviruses (HBoVs) 1–4 are recently discovered, antigenically similar parvoviruses. We examined the hypothesis that the antigenic similarity of these viruses could give rise to clinically and diagnostically important immunological interactions. IgG and IgM EIAs as well as qPCR were used to study ~2000 sera collected from infancy to early adolescence at 3–6-month intervals from 109 children whose symptoms were recorded. We found that HBoV1-4-specific seroprevalences at age 6 years were 80%, 48%, 10%, and 0%, respectively. HBoV1 infections resulted in significantly weaker IgG responses among children who had pre-existing HBoV2 IgG, and vice versa. Furthermore, we documented a complete absence of virus type-specific immune responses in six viremic children who had pre-existing IgG for another bocavirus, indicating that not all HBoV infections can be diagnosed serologically. Our results strongly indicate that interactions between consecutive HBoV infections affect HBoV immunity via a phenomenon called “original antigenic sin”, cross-protection, or both; however, without evident clinical consequences but with important ramifications for the serodiagnosis of HBoV infections. Serological data is likely to underestimate human exposure to these viruses.

## Introduction

Human bocavirus (HBoV) 1 is a commonly circulating human parvovirus associated with upper respiratory tract illness (URTI). While HBoV1 acute infection often is diagnosed by PCR analysis of respiratory samples, the interpretation of such results is complicated. The line between acute and past infection is blurred by a prolonged presence of the viral DNA in some individuals, up to several months after initial infection [1–5]. Detection of HBoV1 mRNA [6, 7], serum DNA [8–11] or anti-HBoV1 antibodies [8, 11, 12] can be considered more reliable tools for the diagnosis of primary infection.

To serodiagnose a HBoV1 infection is, however, not without caveats, due to the circulation of three other closely related human bocaviruses, HBoV2-4 [13, 14]. In contrast to HBoV1, these viruses occur very infrequently in respiratory specimens and appear to be enteric [15]. HBoV1-4 are structurally similar with a difference in amino acid sequence of only 10–20% within the major structural component, viral protein 2 (VP2). This similarity manifests as serological cross-reactivity [16, 17] and may give rise to “original antigenic sin” (OAS), a long-known phenomenon where a prior infection by a virus inhibits the immune response towards the unique epitopes of a subsequent related virus [18], which could affect the clinical outcome. These features have to be taken into consideration in serological assays.

Published studies assessing the clinical significance of HBoV2-4 have so far been limited to nucleic acid-based approaches [4, 13, 19, 20]. However, the occurrence of HBoV2-4 in stool may be prolonged similarly to many other enteric viruses [21] and to HBoV1 in the respiratory tract [1–3, 22]. This would render PCR positivity inadequate as proof of acute primary HBoV2-4 infection and highlight the importance of using serology (or possibly mRNA detection) in clinical assessment.

The main objectives of this study were to assess the epidemiology and immunology of HBoV infections, and to assess their clinical significance. To this end, we used IgG and IgM EIAs as well as qPCR to analyze sera collected from constitutionally healthy children at 3- to 6-month intervals from infancy up to adolescence. Our results reveal new aspects of HBoV epidemiology by showing that HBoV seroprevalences are likely to downplay the proportion of people who have experienced infection by more than one bocavirus type. This is due to novel interactions in humoral responses to HBoV infections, however, with no apparent clinical consequences.

## Methods

### Ethical Statement

The ethics committee of the Hospital District of Southwest Finland approved the study protocol. The legal guardians of the study participants provided written informed consent. All clinical investigation was conducted according to the principles expressed in the Declaration of Helsinki.

### Patients and Serum Samples

The participants were derived from the ongoing population-based Type 1 Diabetes Prediction and Prevention (DIPP) study, a birth cohort study monitoring preclinical events preceding type 1 diabetes among genetically susceptible children in Finland (for details see [23, 24]). Of these children, 109 have previously been analyzed from infancy up to early adolescence by HBoV1 non-competitive immunoglobulin (Ig)M, IgG, and IgG-avidity enzyme immunoassays (EIA) and quantitative PCR (qPCR) [8, 12]. We analyzed 1,943 follow-up serum samples from the same 109 children (median 17 samples per child, mean 18, range 12–27) followed from a median age of 4 months (mean 3 mo, range 2–11 mo) to a median of 8 years (mean 8 y, range 4–13 y). The children were sampled at a median interval of 96 days (mean 110 d, range 55–484 d) until 2 years of age and subsequently at a median interval of 182 days (mean 197 d, range 92–849 d) until October 2008 (unless discontinued earlier). Ages shown hereafter were calculated according to the midpoints of the follow-up sampling intervals.

At each child’s scheduled visit, the parents completed a questionnaire and were interviewed by a study nurse on any clinical symptoms or illnesses since the previous visit. In our previous study of this same cohort of children, the symptoms were classified by a single co-author. To increase the accuracy of data interpretation for the present study, the symptoms were re-classified jointly by a group of three co-authors. Acute otitis media (AOM), sinusitis, tonsillitis and lower respiratory tract infection (LRTI) were diagnosed by a physician. While diabetes-associated autoantibodies developed in 7 (6%) children, none progressed to clinical diabetes.

All serum samples were tested for HBoV1-3 IgG with and without competition with soluble virus-like particles (VLPs), to account for serological cross-reactivity [16]. In the case of the rare HBoV4 IgG [16, 17], only the midmost and last samples from each child were tested. HBoV1-3 IgM and HBoV1-4 quantitative PCR (qPCR) assays were conducted on all specimens showing primary or secondary IgG increases (in non-blocked EIA) and the two specimens flanking these increases.

PCR was further done for 44 stool samples available from the 109 children, consecutively obtained up to 6 months post-infection. Of these 44 stool samples, 8 were from children with HBoV1 and 4 from children with HBoV2 infection.

### Enzyme Immunoassays

Expression and purification of recombinant VLP antigens for HBoV1-4 was performed as described [16]. IgG EIAs were conducted as described [12] except that biotinylated VLPs in the solid phase were used at 175 ng/well, bovine serum albumin was included in the antigen dilution buffer at 25 μg/ml and the substrate incubation time was increased to 15 minutes. IgM EIAs were carried out as described [12].

For the detection of HBoV1-specific antibodies, we competed serum samples with HBoV2 and -3 VLPs (30 μg/ml in PBS, 1.5 h, +4°C), and then tested the sera on plates with immobilized HBoV1 antigen.

For preliminary detection of HBoV2 and -3 antibodies, we performed competition of the serum samples only with HBoV1 antigen before the EIAs. Seroconversion samples, with IgG towards both viruses, and the last follow-up sample of each child, were reanalyzed with double competition (HBoV1+3 or HBoV1+2). The final HBoV2 and -3 seroprevalences were designated based on the double-competition assays.

Absorbances measured in these competition assays yielded the raw optical densities (OD). Successful competition was confirmed by parallel analysis with homologous competing antigen, yielding the residual ODs. The residual ODs were typically very low (median 0.03; 90th percentile, 0.11). To avoid false positive results due to incomplete antibody competition, the net OD was calculated by subtracting the residual OD from the raw OD. Competed samples were designated antibody positive or negative based on the net OD.

To determine the HBoV1 IgM-EIA cutoff, we selected serum samples from children aged >8 years on the basis that HBoV1 primary infections are highly uncommon in this age group [8]. Samples with major (>0.5 OD) fluctuations in IgG levels in the neighboring follow-up samples were excluded, yielding 138 samples from 27 children. The cutoffs (mean + 4 SDs) were 0.131 and 0.092 for the respective non-competed and competed IgM assays.

For IgG-EIA cutoff determination we selected samples (n = 111) preceding HBoV1 IgM seroconversion or HBoV1 viremia. Cutoff absorbances (mean + 4 SDs) for positive competed and non-competed IgG EIA results were 0.151 and 0.095, respectively. The HBoV1 IgG- and IgM-EIA cutoffs were applied to HBoV2-4 EIAs as such based on the uniformity of the protein purification and EIA methods and due to inadequate number of HBoV2-4 control sera.

Unless explicitly stated, all references to the occurrence of IgG or IgM in the following text and Tables 1–3 are based on the virus-specific competition assays, or such non-competition assays where only a single virus showed a positive IgG or IgM result.

**Table 1:** HBoV1-3 IgG stability among 109 constitutionally healthy children.

	HBoV1	HBoV2	HBoV3
IgG+	94	58	11
High & stable IgG^a^	43 (46%)	0	0
Substantial IgG decrease^b^	8 (9%)	21 (36%)	4 (36%)
Secondary IgG increase^c^	2 (2%)	22 (38%)	5 (45%)
Remaining children	41 (44%)	15 (26%)	2 (18%)

Abbreviations: HBoV1, human bocavirus 1; HBoV2, human bocavirus 2; HBoV3, human bocavirus 3; IgG, immunoglobulin G

^a^ Competed IgG showed no significant waning and remained above 2.0 absorbance units from initial seroconversion to the end of the follow-up period

^b^ ≥2 absorbance unit decrease in optical density after seroconversion

^c^ ≥2 absorbance unit increase in optical density in two consecutive samples, both obtained at least 4 months after IgG seroconversion

Note: the percentages do not necessarily add up to 100% because of rounding up.

**Table 2:** Co-occurrence of IgM or viremia with human bocavirus 1, 2 or 3 primary IgG seroconversions among 109 constitutionally healthy children.

	IgG seroconversion	Viremia	IgM	IgG seroconversion with viremia	IgG seroconversion with IgM	IgG seroconversion with viremia and IgM
HBoV1	94	24	35	22	32	10
HBoV2	58	8	11	4	10	2
HBoV3	11	1	0	1	0	0

Note: data are no. of patients. Viremia or IgM was regarded to coincide with IgG seroconversion if they were present in the first IgG positive sample or the sample preceding this sample. HBoV4 is excluded from the table due to absence of detectable infections. Abbreviations: HBoV, human bocavirus; IgG, immunoglobulin G; IgM, immunoglobulin M.

**Table 3:** Infection-related symptoms during human bocavirus 1 and 2 primary seroconversions compared with those of the previous and subsequent sampling intervals.

	Primary HBoV1 immune responses, n = 64					Primary HBoV2 immune responses, n = 44				
	SC interval	previous interval		next interval		SC interval	previous interval		next interval	
Symptom	no. (%)	no. (%)	p value	no. (%)	p value	no (%)	no. (%)	p value	no. (%)	p value
URTI	29 (45)	31 (48)	0.86	20 (31)	0.12	13 (30)	14 (32)	1.0	17 (39)	0.50
Gastroenteritis	14 (22)	11 (17)	0.66	10 (16)	0.50	14 (32)	6 (14)	0.08	8 (18)	0.24
Acute otitis media	31 (48)	20 (31)	0.04*	23 (36)	0.20	12 (27)	6 (14)	0.18	12 (27)	1.0

Note: first HBoV1 seroconversion was regarded as primary regardless of the presence or absence of pre-existing HBoV2 or -3 IgG, and vice versa. SC, seroconversion; URTI, upper respiratory tract illness; HBoV1, human bocavirus 1; HBoV2, human bocavirus 2.

Boldface and * indicate statistical significance by Liddell exact test (<0.05).

## Results

### IgG Seroprevalence

**Fig 1:**
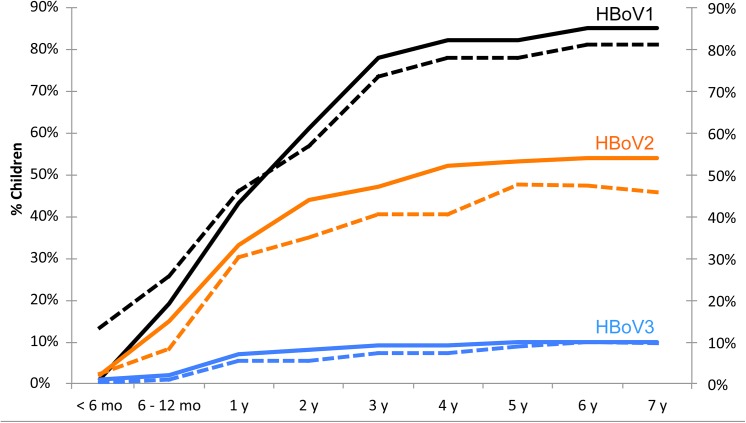
Human bocavirus 1–3 seroprevalences (dashed lines) and cumulative seroconversion rates (solid lines) in different age groups.

HBoV1-3 seroprevalences as a function of age are illustrated in Fig 1. Individual ELISA results of all 109 children are illustrated graphically in S1 Supporting Information. All children seroconverted for at least one HBoV by the age of 4.8 y. The median age of seroconversion for those who seroconverted within the follow-up, was 1.9 years (range 0.5–8.0) for HBoV1, 1.6 years (range 0.4–9.3) for HBoV2 and 1.7 years (range 0.4–5.8) for HBoV3. By the age of 6 years, among those 93 (85%) children who still remained in follow-up, the seroprevalences for HBoV1, 2 and 3 were 80%, 48% and 10%, respectively. No HBoV4 IgG was detected in any of the children.

HBoV1-3 IgG reversions were evident in 10/94 (11%), 13/58 (22%) and 3/11 (27%) seropositive children, respectively. These IgG reversions were due to weak IgG responses that waned below cutoff over time (e.g. child #13 in S1 Supporting Information). Of the IgG-reverted children, one-third later regained HBoV1 IgG and two-thirds HBoV2 IgG.

### IgG Stability

**Fig 2:**
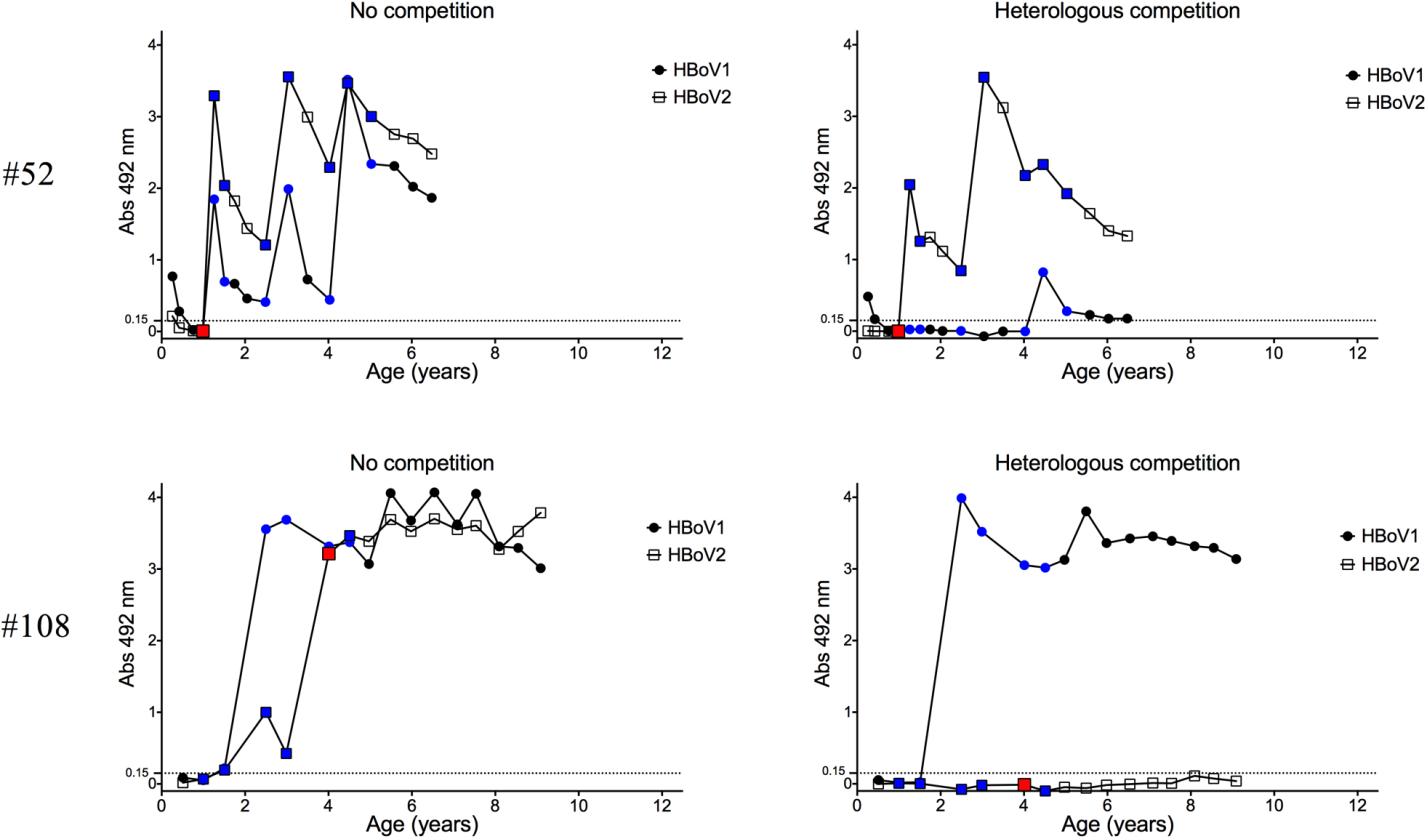
Pre-existing HBoV1 IgG reduces immune response against HBoV2 infection. Two representative cases of PCR-verified HBoV2 infections without (child #52) or with (child #108) pre-existing HBoV1 IgG are shown. The red, blue and black data points respectively indicate whether the serum tested positive by PCR, tested negative by PCR, or was not tested by PCR. Although not shown in the graphs, both children tested negative for HBoV3- and 4-specific IgG.

There was significant variability in HBoV1-3 IgG OD stability between individual children. Typically, however, HBoV1 IgG levels remained higher and more stable than those of HBoV2 (Tables 1). The latter were characterized by repeated sharp increases and decreases (e.g. subject #52 in Fig 2). Based on the limited data, HBoV3 IgG profiles resembled those of HBoV2.

### IgG Co-Occurrence

HBoV2 IgG occurred more rarely in patients with HBoV1 IgG than without, and vice versa. All 15 subjects without, but only 43 of 94 (46%) subjects with HBoV1 IgG showed also HBoV2 or -3 IgG. Similarly, all 51 subjects without, but only 43 of 58 (74%) with HBoV2 or -3 IgG, showed also HBoV1 IgG. Both results were statistically highly significant (p<0.001) by Fisher's exact test. HBoV3-positive subjects were too few for meaningful statistical analysis.

**Fig 3:**
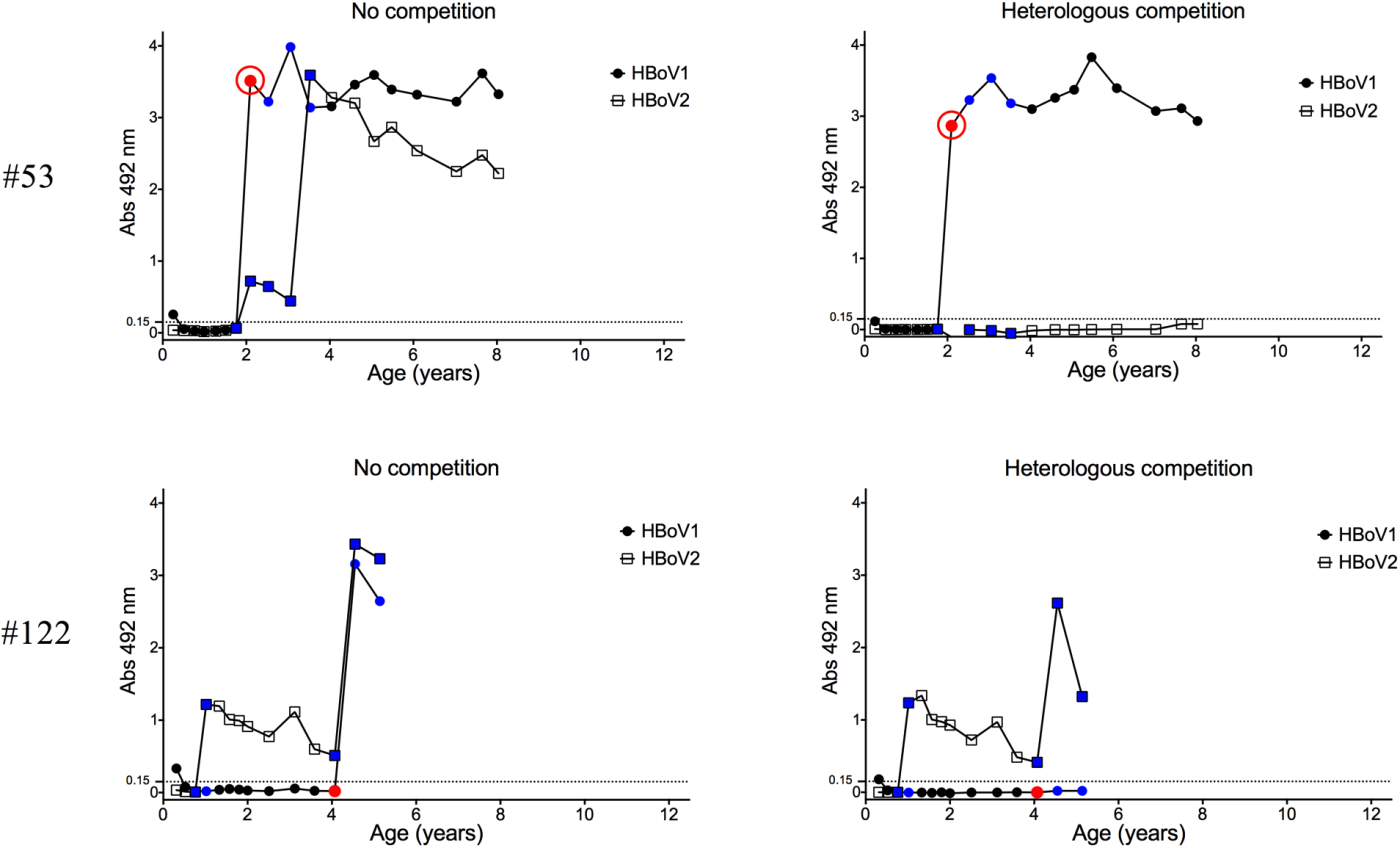
Pre-existing HBoV2 IgG reduces immune response against HBoV1 infection. Two illustrative cases of PCR-verified HBoV1 infections without (child #53) or with (child #122) pre-existing HBoV2 IgG are shown. The red, blue and black data points respectively indicate whether the serum tested positive by PCR, tested negative by PCR, or was not tested by PCR. The red open circle indicates a sample that was positive for HBoV1 IgM. Although not shown in the graphs, both children tested negative for HBoV3- and 4-specific IgG.

The presence of HBoV2 or -3 IgG antibodies preceding HBoV1 IgG was also inversely associated with HBoV1 IgG levels. Only 8 of 22 (36%) children with HBoV2 or -3 IgG preceding HBoV1 seroconversion, developed moderate or high HBoV1 IgG absorbances (> 1.0) in contrast to all 68 non-primed children (p<0.0001 by Fisher’s exact test). Subjects #53 and #122 in Fig 3 demonstrate HBoV1 responses in children without or with pre-existing HBoV2 IgG, respectively. Likewise, the presence of HBoV1 IgG prior to HBoV2 seroconversion was associated with low IgG reactivity toward the latter. Of 17 children with pre-existing HBoV1 IgG, only 7 (41%) developed moderate to high HBoV2 IgG absorbances as opposed to all 37 (100%) non-primed children. Subjects #52 and #108 in Fig 2 demonstrate HBoV2 responses in children without or with pre-existing HBoV1 IgG, respectively.

Waning maternal IgG against HBoV1 was detected in 51%, and against HBoV2 or HBoV3 in 7%, of 88 children from whom the first sample was taken within the first 6 months after birth. As with primary infections, the maternal HBoV1 IgG levels were more pronounced than those of HBoV2.

### IgM

IgM for HBoV1 or -2 was detected almost exclusively in conjunction with a seroconversion for the corresponding (competed) IgG (Tabel 2). Only 3 subjects showed HBoV1 IgM in more than one follow-up sample, potentially due to nonspecific reactivity. All HBoV2 IgM reactivities lasted <6 months after seroconversion. No HBoV3 IgM was observed.

Detection of HBoV1 IgM was very rare in children who already were seropositive for HBoV2 or -3. It occurred in 32 of 61 (52%) children in whom HBoV1 was the first infecting HBoV but only in 2 of 48 (4%) children primed with HBoV2 or -3.

### Reassessment of HBoV1 Serology

We have previously studied this same cohort with HBoV1 EIA assays without competition [8] and found that all the 109 children became IgG seropositive by 6 years of age. The present HBoV1 IgG results are, based on the new competition EIA, notably different; 15 of the 109 children showed no detectable HBoV1 IgG at any time point but instead showed HBoV2 or -3 IgG. Further 12 children experienced seroconversion of HBoV1-specific IgG at a later time point than determined by the non-competition assay (exemplified by subject #52 in Fig 2). On the other hand, the effect of HBoV2 and HBoV3 circulation on our HBoV1 IgM results appears to have been minimal, with only one of the 39 previously observed HBoV1 IgM positives [8] being apparently due to the enteric bocaviruses.

### Real-Time qPCR

The occurrence of HBoV1 viremias in this cohort of 109 children has been determined previously [8]. Of the 94 HBoV1-infected children, 24 (26%) were viremic around the time of infection, however, none of the 27 children with a previous false HBoV1 seroconversion had viremia at that time. HBoV2 or -3 viremia was detected respectively in four of 58 (7%) and one of 11 (9%) children with corresponding IgG conversion. Of note, none of the children showed repeated viremia by any given bocavirus type.

Almost all HBoV1 viremias were observed concurrently with a seroconversion for (competed) HBoV1 IgG (Table 2). HBoV2 or HBoV3 IgG preceded the HBoV1 viremia in five of the 24 viremic cases. Two of these five HBoV1 viremias were not associated with observable production of HBoV1 IgG in competition EIA, even though significant increases in HBoV1 IgG were seen in the non-competition EIA (e.g. child #122 in Fig 3).

In contrast to HBoV1, only 4 of the 8 HBoV2 viremias were associated with a concurrent primary IgG seroconversion for the virus. None of these 4 seroconverting children showed pre-existing antibodies for any bocavirus. The remaining 4 children were primed with HBoV1 (n = 3) or HBoV3 (n = 1) IgG before the appearance of HBoV2 viremia and showed no HBoV2 IgG responses; one such case is illustrated in Fig 2 (subject #108). To confirm these results, the serum DNA of each child was re-isolated from a separate aliquot. All 8 detections of HBoV2 viremia were found to be reproducible.

For HBoV1, the DNA loads in the 5 viremic children primed with HBoV2 or -3 IgG were 1.3×10^0^–2.2×10^4^ copies/ml, whereas those for the 19 non-primed viremic children were 5.3×10^3^ to 9.1×10^5^ copies/ml. Furthermore, all HBoV2 (n = 8) and HBoV3 (n = 1) viral loads (<7×10^3^ copies/ml) were even lower, near the limits of reliable qPCR detection. The numbers of children and the virus loads were too low for meaningful statistical analysis of the possible influence of prior immunity on secondary heterologous virus replication.

No child tested DNA positive for multiple bocavirus species in any given sample. Altogether, only one child (#5 in S1 Supporting Information) tested viremic more than once; for HBoV3 DNA at the age of 3 months and for HBoV2 at 4.1 years. The child had also HBoV1 IgG, thus representing the only case of a reasonably verified triple HBoV infection in the cohort, serologically visible only in the non-competition EIA as three successive IgG peaks.

Half of the 8 HBoV1-infected children with consecutive stools showed HBoV1 DNA in the first stool 0.5–1.1 months after seroconversion but not later. In contrast, all 4 HBoV2-infected children with consecutive stools had HBoV2 DNA in the first stool, and 3 had in several stools, lasting 1,2 or 5 months after seroconversion.

### Clinical Correlates

By comparing signs and symptoms during the seroconversion (SC) interval with those during the flanking intervals of each child, a statistically significant association (p<0.05 by Liddell exact test) was observed between HBoV1 and AOM (p = 0.04, for one of the two flanking intervals) but not for the other two common illnesses URTI and gastroenteritis (Table 3). Other symptoms (LRTI, fever, tonsillitis, conjunctivitis, sinusitis & exanthema) were observed too rarely for meaningful statistical analyses (data not shown). The severity of symptoms reported during secondary HBoV1-infection intervals, and their flanking intervals, did not markedly differ in the 15 children lacking specific secondary IgG responses, compared with the children exhibiting a robust secondary immune response. The HBoV2-infected children showed more gastrointestinal symptoms during the infection interval than in the flanking intervals, without reaching statistical significance (Table 3). The low number of children prevented symptom analysis of HBoV3 and -4 infections.

## Discussion

We have previously provided evidence to indicate that most HBoV1 infections are serologically specific even without antigen competition in the acute infection phase [16]. However, two aspects of this notion should be clarified. First, specificity depends heavily on the experimental methodology. Our current non-competition EIAs with elevated antigen loads do show cross-reactivity in some acute-phase patients with high antibody levels. Second, HBoV1 IgG seroconversion in a non-competition assay by itself is not a definite marker of an acute HBoV1 infection because the seroconversion can be due to a rise in HBoV2-4 IgG.

In our earlier assessment of the etiological role of HBoV1 in these same 109 constitutionally healthy children [8], we nevertheless used non-competition-IgG seroconversion as the prime marker of acute infection. We found that acute HBoV1 infection was strongly associated with upper respiratory tract infection and less with acute otitis media. However, the present reassessment of HBoV1 clinical correlates, with revised methodology, surprisingly showed only a weak correlation with acute otitis media and none with respiratory illness. Reasons for the discrepancy might be that the new procedure yielded a different set of HBoV1-positive subjects resulting in 64 vs. 101 children, and that the URTI symptoms in 38 children were classified differently upon blinded re-evaluation by a group of three co-authors. Despite the uniquely high serum sampling coverage, the intervals were possibly still too long for significance assessment among children of this age, with several URTIs a year.

We have previously hypothesized that, in contrast to the systemic HBoV1 infections, infections by the enteric bocaviruses would be local and result in weak immune reactions [16]. This notion was based on the less frequent detection of HBoV2 or HBoV3 IgG among adults than children, on the low level of IgG in seropositive adults, and on the lack of viremia in children. Our present data show that in some children primary HBoV2 and HBoV3 infections can present with markers of systemic infection, i.e. viremia and a strong IgG response. Nevertheless, on the whole HBoV2 and possibly HBoV3 viremias appear to be rare, low-titered, and/or short-lived, and the corresponding IgG responses are generally weaker and more prone to waning than those of HBoV1. While boosts of these initially low HBoV2 and -3 IgG levels appear commonly, they do not seem to prevent the IgG from waning. Even if our pediatric follow-up period did not extend beyond the early teens, the very low incidence of maternal HBoV2 or -3 IgG in the infants supports our previous observation of low (or undetectable) IgG levels in adults [16].

We observed several PCR-verified heterologous secondary infections that failed to stimulate significant production of IgG specific for the boosting virus. Instead, the immunostimulatory effect, visualized by non-competition EIAs, preferentially targeted epitopes shared with the priming virus. We also noted that when the secondary (type-specific) IgG responses did emerge, they were typically weak in comparison to the corresponding primary IgG responses. Furthermore, HBoV1 IgM responses were almost completely absent from individuals with prior HBoV2 or -3 IgG, as compared to half of those with HBoV1 as the first HBoV infection. These results are in line with the proposed phenomenon of original antigenic sin (OAS), i.e. the propensity of the body’s immune system to become primed by an antigen and subsequently disregard, partially or completely, the novel epitopes of related subsequent antigens [18, 25, 26].

The OAS concept was originally formulated by influenza virus researchers in the 1960s and has since been associated with several other viruses, including dengue viruses (DENV) as a frequently cited example [27–29]. In DENV infections, severe symptoms such as high fever and shock from increased vascular permeability appear to occur more often in secondary than in primary infections [30, 31]. The aggravated DENV infections may involve OAS preventing the formation of neutralizing antibodies against novel epitopes. The subsequent circulation of poorly neutralizing cross-reactive antibodies may assist infection by otherwise noninfectious immature DENV particles via Fcγ receptor-bearing hosts such as macrophages in a phenomenon known as antibody-dependent enhancement of infection (ADE). The latter has been observed not only with DENV [32, 33] and influenza viruses [34, 35] but also with parvoviruses such as Aleutian mink disease parvovirus and human parvovirus B19 [36–39].

OAS is generally perceived as a detrimental phenomenon that may clinically aggravate infections or expose a host to repeated infections. In our clinical evaluation, however, no such effects were evident. Unfortunately, the occurrence of PCR-verified OAS was too infrequent for statistically relevant analysis. On the other hand, despite the existence of such viremias, our data could perhaps be explained by cross-protection, whereby pre-existing immunity against one bocavirus limits the propagation of another. This could prevent the secondary antigen from reaching the quantity threshold required for efficient naïve B-cell activation. In other words, the absence or weakness of the secondary responses could reflect the virtue of an efficiently functioning primary immune memory rather than the sin of an imperfect secondary response. Indeed, we are not sure to what extent existing OAS literature could be explained by immunological cross-protection rather than a "blind spot" in the immune system. However, we have recently studied immune responses in rabbits sequentially immunized with recombinant HBoV1-4 VLPs and observed muted antibody responses against the secondary antigens in several animals [40]. Considering that the animals were exposed to hundreds of micrograms of antigen (as opposed to minute doses of live viruses), it seems unlikely that the diminished secondary antibody responses could be explained solely by antigen elimination by a cross-reactive immune system.

Regardless of the clinical significance or the exact mechanism of weak B-cell responses to secondary (heterologous) HBoV1-4 infections, our results have important diagnostic ramifications for the serodiagnostic assessment of HBoV infections. We documented six cases of HBoV viremia that did not result in an observable species-specific antibody response against the viremic species. Such data indicate that most but not all HBoV infections can be diagnosed serologically. More such viremic cases in our study may have gone unrecognized due to the long sampling intervals (3–6 months) and selective PCR testing of the sera. This indicates that the seroprevalences obtained here and elsewhere for HBoV1-4 are, due to both IgG waning and OAS, undoubtedly underestimations of the extent to which people are exposed to these viruses. For comparison, a relatively recent DENV study illustrated the persisting difficulty of serologically diagnosing secondary DENV infections, despite these viruses having been known for decades. By using serum PCR as "gold standard", the authors were able to infer the correct secondary DENV serotype with 67% accuracy based on pre- and postinfection neutralizing antibody titers [41]. Highly accurate serologic differentiation of secondary HBoV infections may prove to be an equally elusive task. Serodiagnosis of HBoV infections nevertheless remains as important as ever since the clinical significance of secondary HBoV infections is yet to be fully assessed.

## Supporting Information

S1 Supporting Information ELISA results of all 109 children in graphical format. (PDF)
