# Correction: B-Cell Responses to Human Bocaviruses 1–4: New Insights from a Childhood Follow-Up Study

**DOI:** 10.1371/journal.pone.0172078

**Published:** 2017-02-08

**Authors:** Kalle Kantola, Lea Hedman, Laura Tanner, Ville Simell, Marjaana Mäkinen, Juulia Partanen, Mohammadreza Sadeghi, Riitta Veijola, Mikael Knip, Jorma Ilonen, Heikki Hyöty, Jorma Toppari, Olli Simell, Klaus Hedman, Maria Söderlund-Venermo

There is an error in the second sentence of the sixth paragraph under the subheading “Enzyme Immunoassays” in the Methods section. The correct sentence is: Cutoff absorbances (mean + 4 SDs) for positive competed and non-competed IgG EIA results were 0.095 and 0.151, respectively.
