# Supplementary material for: B-Cell Responses to Human Bocaviruses 1–4: New Insights from a Childhood Follow-Up Study
Source: PLoS One. 2015 Sep 29;10(9):e0139096. doi: 10.1371/journal.pone.0139096 (PMC4587975; doi:10.1371/journal.pone.0139096)
Supplement: S1 Supporting Information — (PDF) [file pone.0139096.s001.pdf]

S1 Supporting Information. ELISA results of all 109 children in graphical format.

Symbol or annotation	Description
2	Subject number. There are three graphs for each subject and a row correspons to one subject.
HBoV1<br>HBoV2<br>HBoV3	Single immobilized antigen used for IgG detection
HBoV2 & HBoV3	Two immobilized antigens used simultaneously for IgG detection.
No competition	Samples were not competed with soluble antigens prior to the assay.
Homologous competition	Samples were preincubated with soluble HBoV1 antigen before HBoV1 IgG detection or with HBoV2+HBoV3 antigens before combined detection of HBoV2 and HBoV3 IgG. The resulting residual optical density (ROD) was used for the calculation of net OD in heterologous competition;<br>net OD = raw OD - residual OD.
Heterologous competition	For HBoV1 IgG detection, the samples were preincubated with soluble HBoV2 and HBoV3 antigens. For HBoV2 or HBoV3 detection, the serum samples were preincubated with HBoV1 antigen. The net OD shown in the graphs was obtained by subtracting ROD from the raw OD;<br>net OD = raw OD - residual OD.
	**Black**, **blue** and **red** data points indicate whether the sample has **not been tested** by PCR for HBoV DNA, has been **negative** by PCR or has been tested **positive** by PCR.
	Red open circle or red open rectangle around a data point indicates that the sample has been positive in IgM for HBoV1 or HBoV2, respectively. All sera tested by PCR for any of the three viruses were tested for HBoV1-3 IgM. IgM negativity is not separately indicated. Red data point surrounded by a red open circle or rectanble indicates that the sample has been positive for IgM and HBoV DNA.
	PCR positive (closed symbol) or IgM positive (open symbol) sample that is below the range of the y-axis.

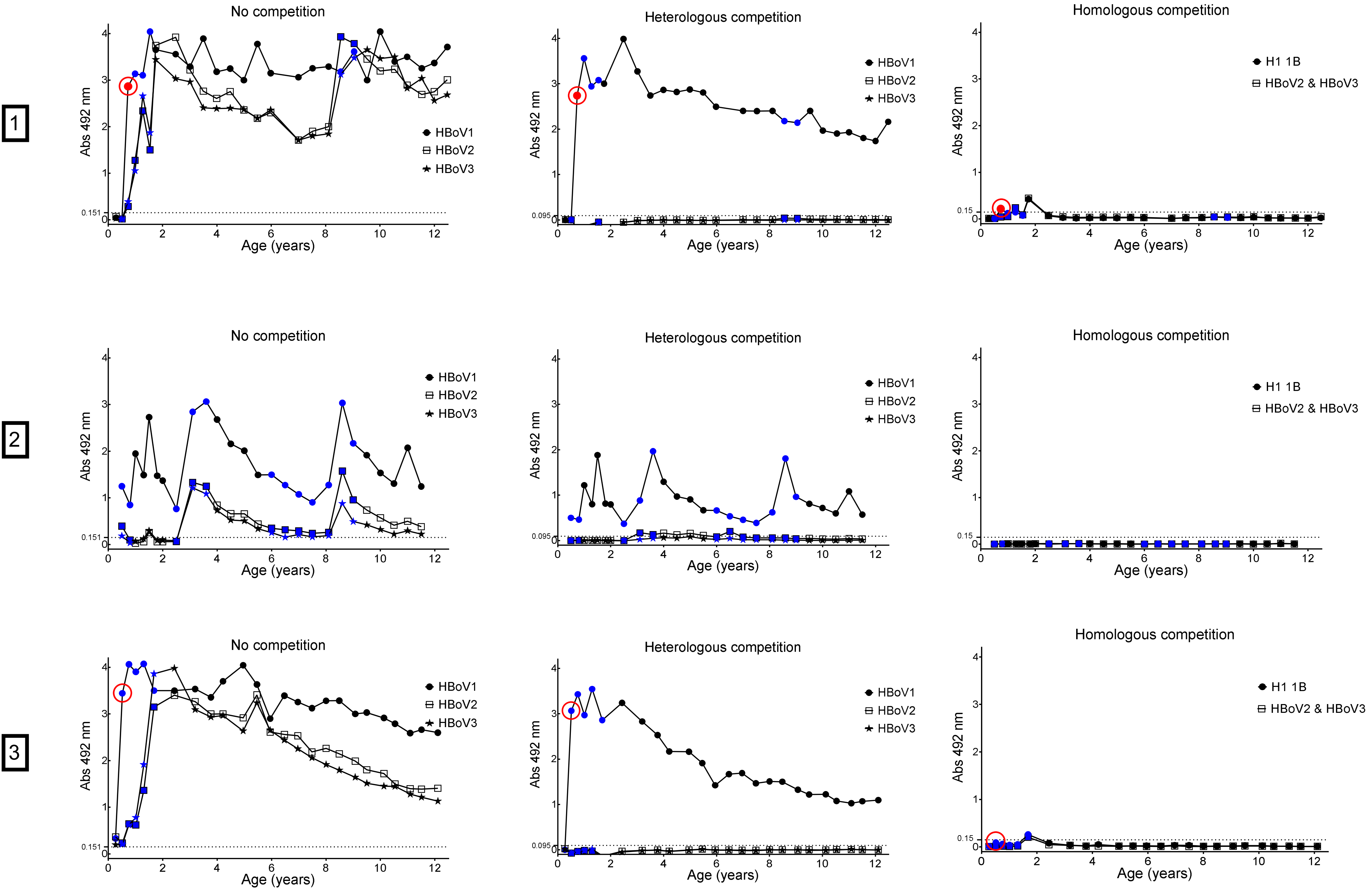
1
2
3
No competition
Heterologous competition
Homologous competition
Abs 492 nm
Age (years)
HBoV1
HBoV2
HBoV3
H1 1B
HBoV2 & HBoV3
0.151
0.095
0.15

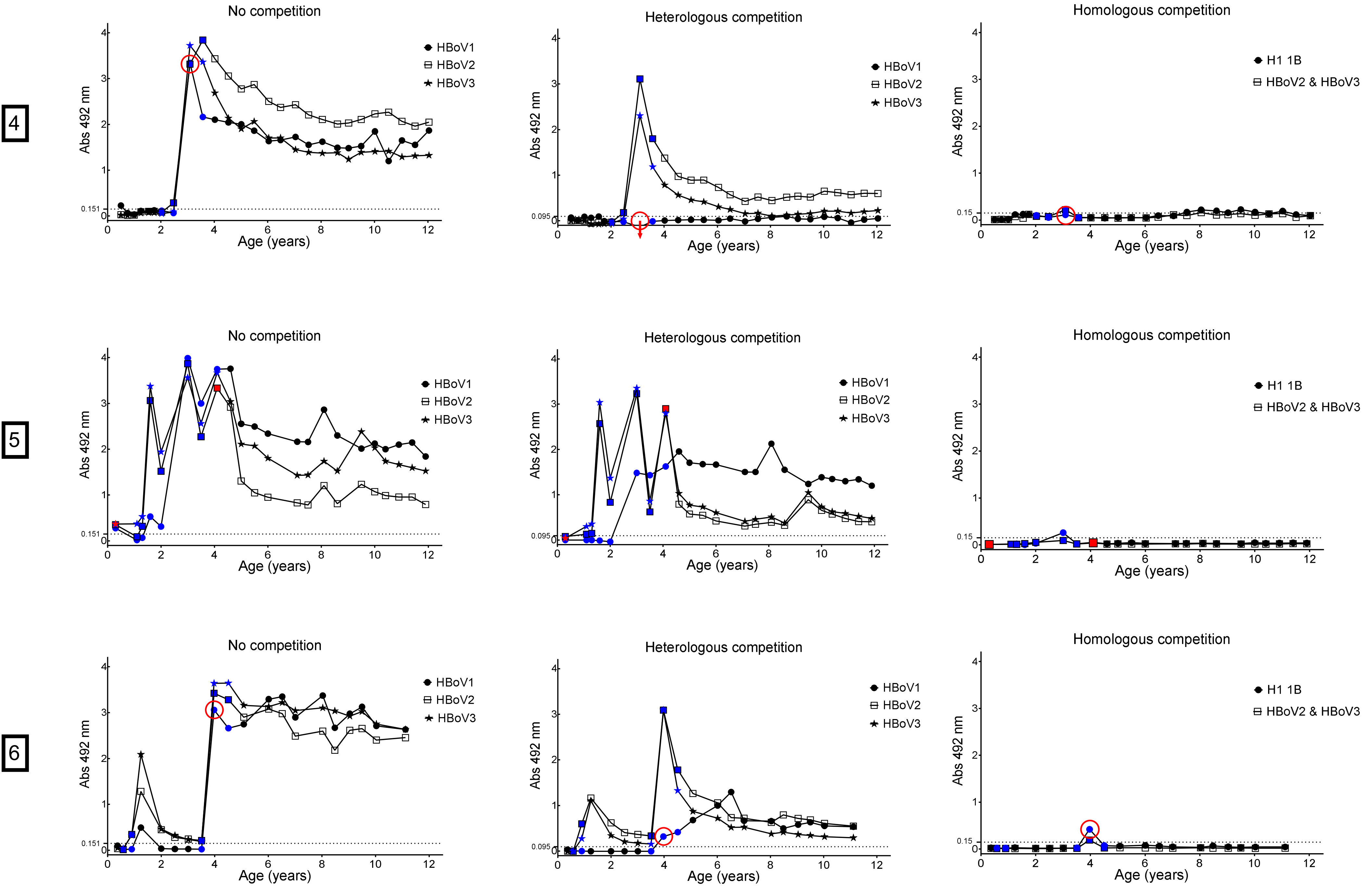
4
5
6
No competition
Heterologous competition
Homologous competition
Abs 492 nm
Age (years)
HBoV1
HBoV2
HBoV3
H1 1B
HBoV2 & HBoV3
0.151
0.095
0.15
0
1
2
3
4
6
8
10
12

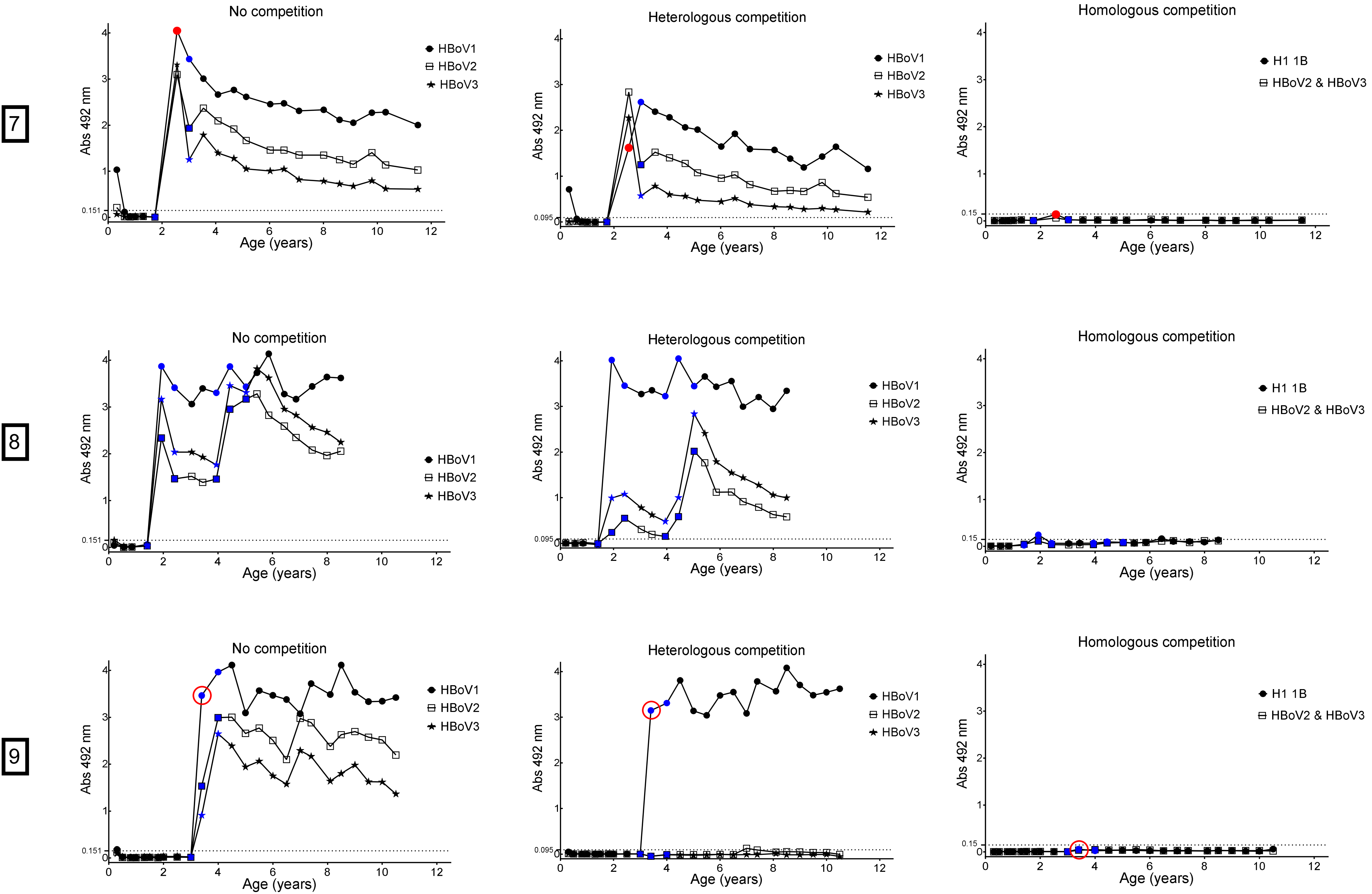
7
8
9
No competition
Heterologous competition
Homologous competition
HBoV1
HBoV2
HBoV3
H1 1B
HBoV2 & HBoV3
Abs 492 nm
Age (years)
0.151
0.095
0.15

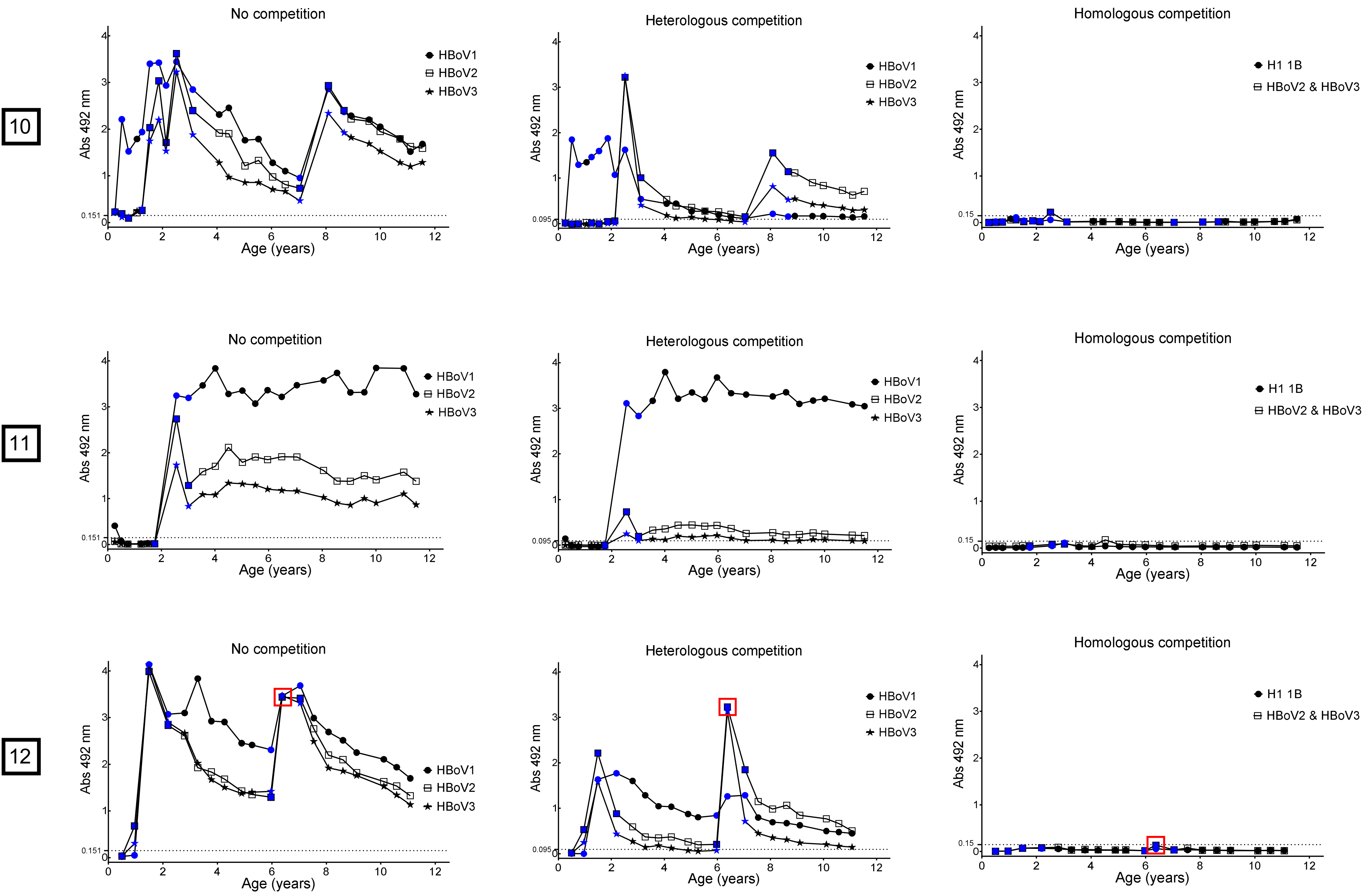
10
11
12
No competition
Heterologous competition
Homologous competition
HBoV1
HBoV2
HBoV3
H1 1B
HBoV2 & HBoV3
Abs 492 nm
Age (years)
0.151
0.095
0.15

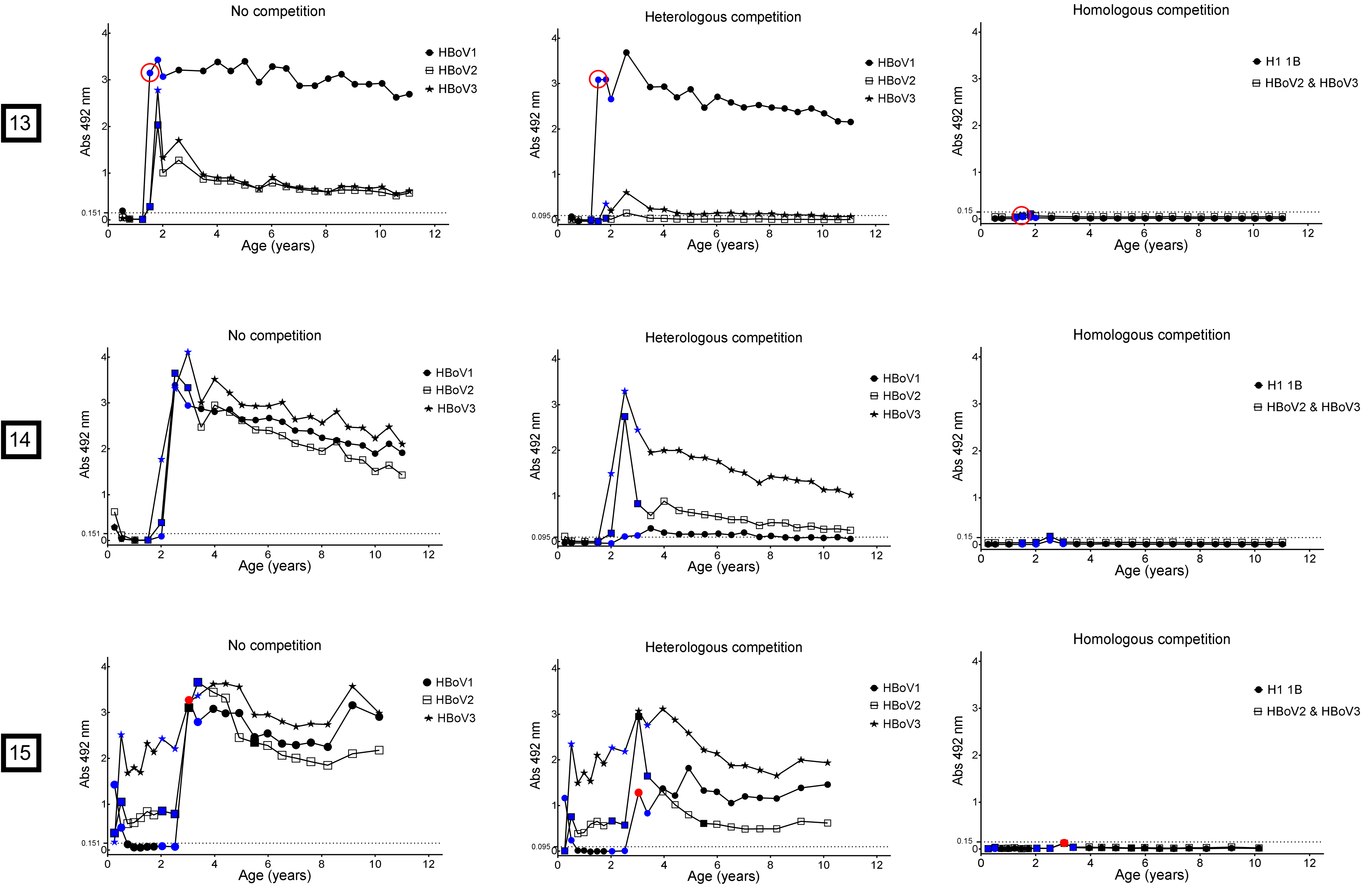
13
No competition
Heterologous competition
Homologous competition
HBoV1
HBoV2
HBoV3
H1 1B
HBoV2 & HBoV3
Abs 492 nm
Age (years)
0.151
0.095
0.15
14
No competition
Heterologous competition
Homologous competition
HBoV1
HBoV2
HBoV3
H1 1B
HBoV2 & HBoV3
Abs 492 nm
Age (years)
0.151
0.095
0.15
15
No competition
Heterologous competition
Homologous competition
HBoV1
HBoV2
HBoV3
H1 1B
HBoV2 & HBoV3
Abs 492 nm
Age (years)
0.151
0.095
0.15

16

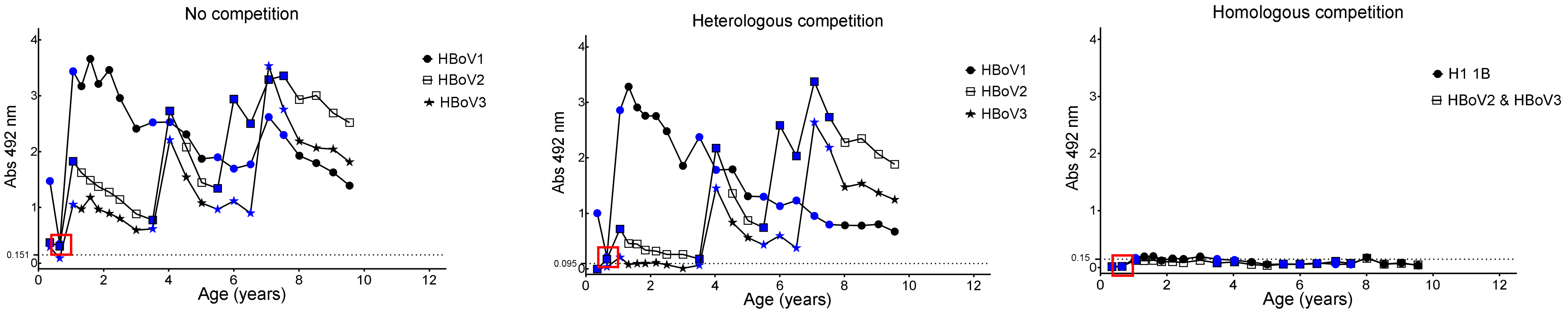
No competition
Heterologous competition
Homologous competition
HBoV1
HBoV2
HBoV3
H1 1B
HBoV2 & HBoV3
Abs 492 nm
Age (years)
0.151
0.095
0.15

17

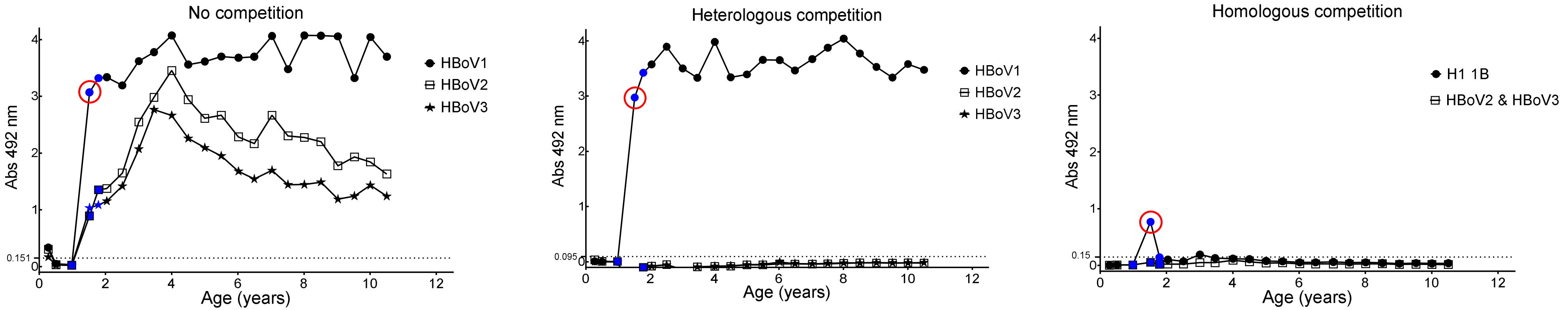
No competition
Heterologous competition
Homologous competition
HBoV1
HBoV2
HBoV3
H1 1B
HBoV2 & HBoV3
Abs 492 nm
Age (years)
0.151
0.095
0.15

18

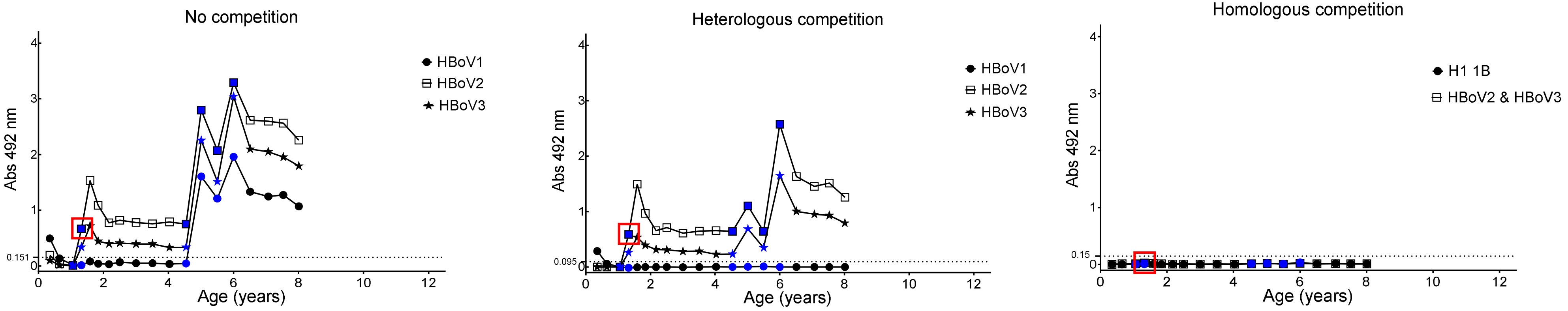
No competition
Heterologous competition
Homologous competition
HBoV1
HBoV2
HBoV3
H1 1B
HBoV2 & HBoV3
Abs 492 nm
Age (years)
0.151
0.095
0.15

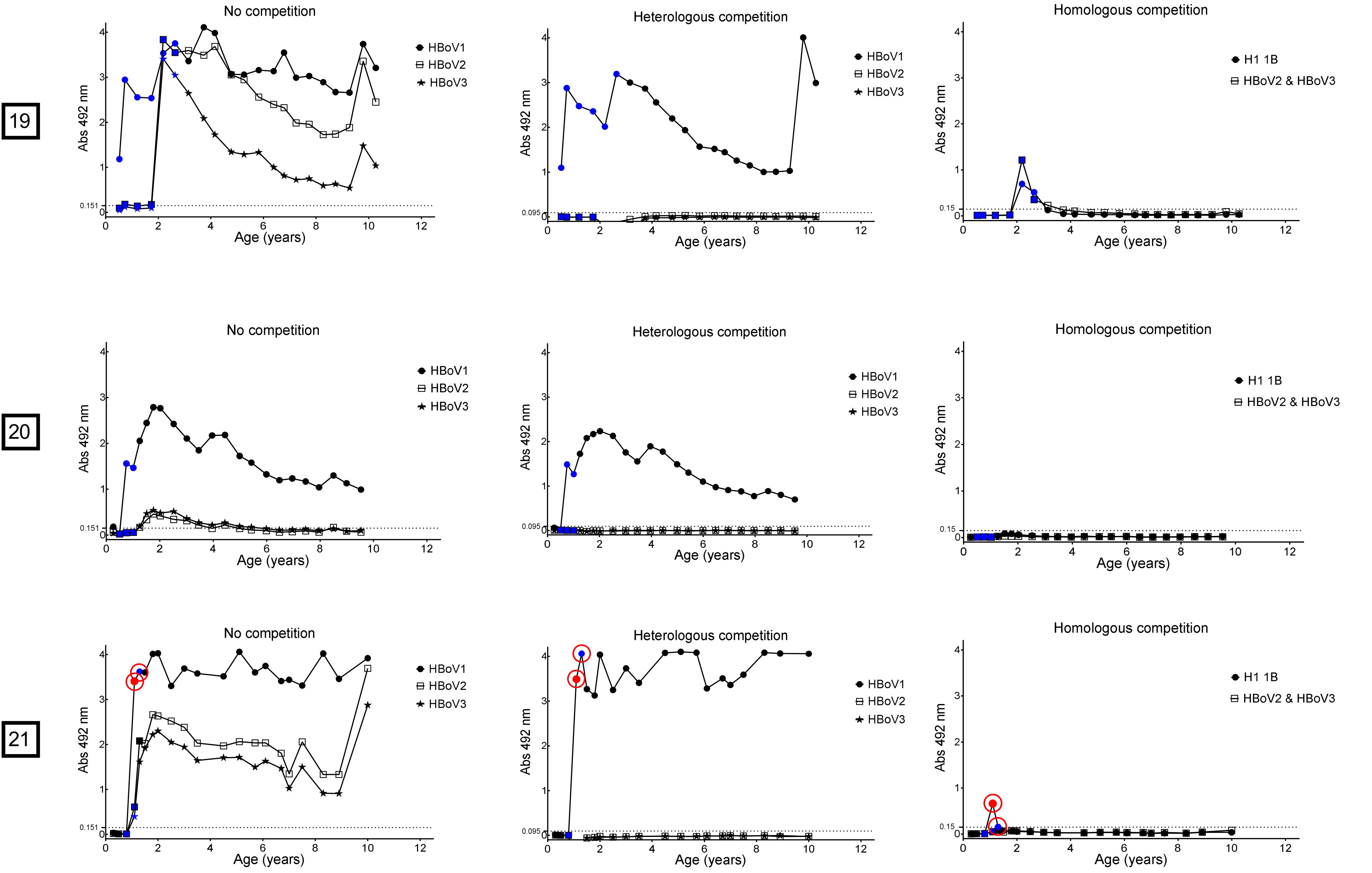
19
No competition
HBoV1
HBoV2
HBoV3
Abs 492 nm
0.151
Age (years)
Heterologous competition
0.095
Homologous competition
H1 1B
HBoV2 & HBoV3
0.15
20
No competition
HBoV1
HBoV2
HBoV3
Abs 492 nm
0.151
Age (years)
Heterologous competition
0.095
Homologous competition
H1 1B
HBoV2 & HBoV3
0.15
21
No competition
HBoV1
HBoV2
HBoV3
Abs 492 nm
0.151
Age (years)
Heterologous competition
0.095
Homologous competition
H1 1B
HBoV2 & HBoV3
0.15

22

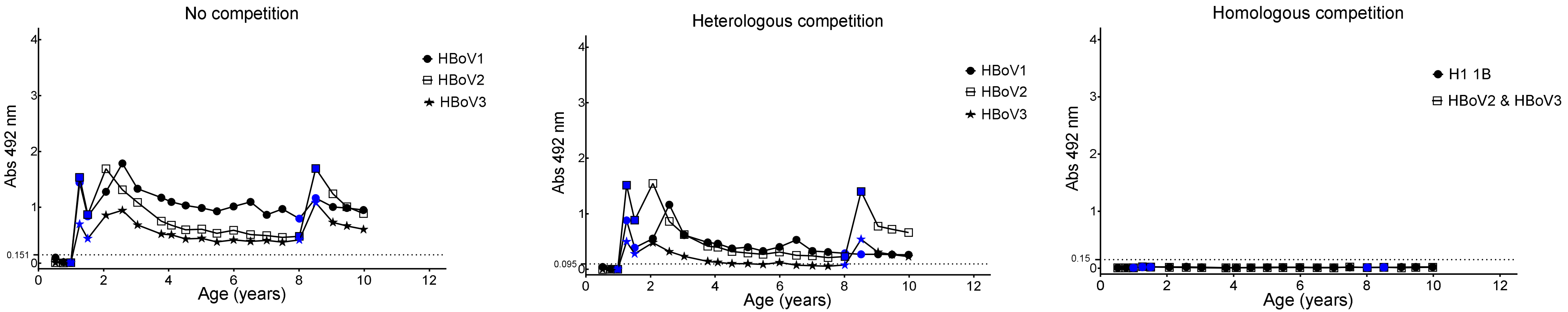
No competition
Heterologous competition
Homologous competition
HBoV1
HBoV2
HBoV3
H1 1B
HBoV2 & HBoV3
Abs 492 nm
Age (years)
0.151
0.095
0.15

23

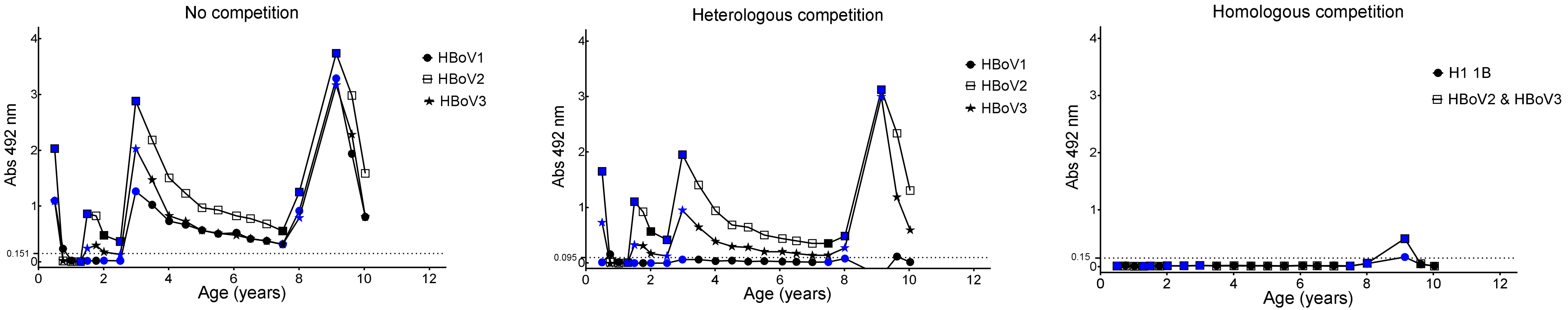
No competition
Heterologous competition
Homologous competition
HBoV1
HBoV2
HBoV3
H1 1B
HBoV2 & HBoV3
Abs 492 nm
Age (years)
0.151
0.095
0.15

24

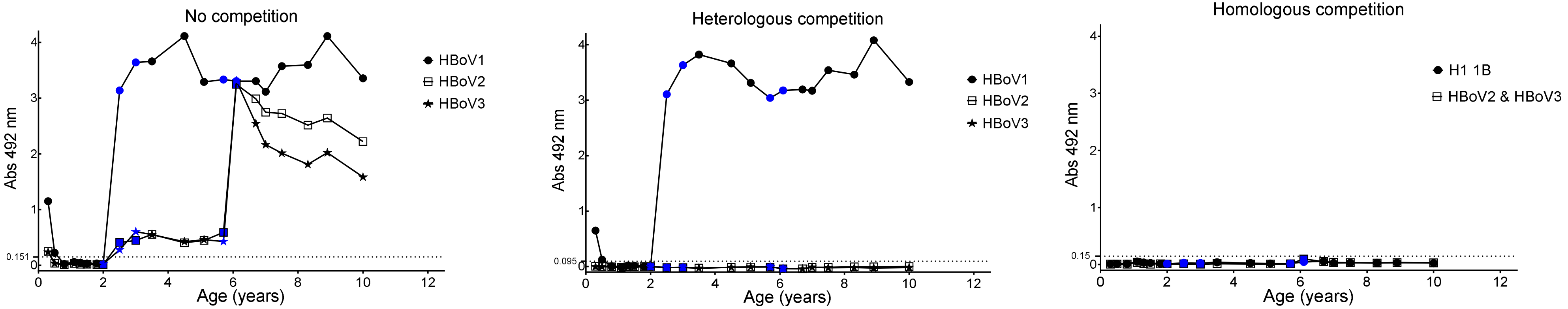
No competition
Heterologous competition
Homologous competition
HBoV1
HBoV2
HBoV3
H1 1B
HBoV2 & HBoV3
Abs 492 nm
Age (years)
0.151
0.095
0.15

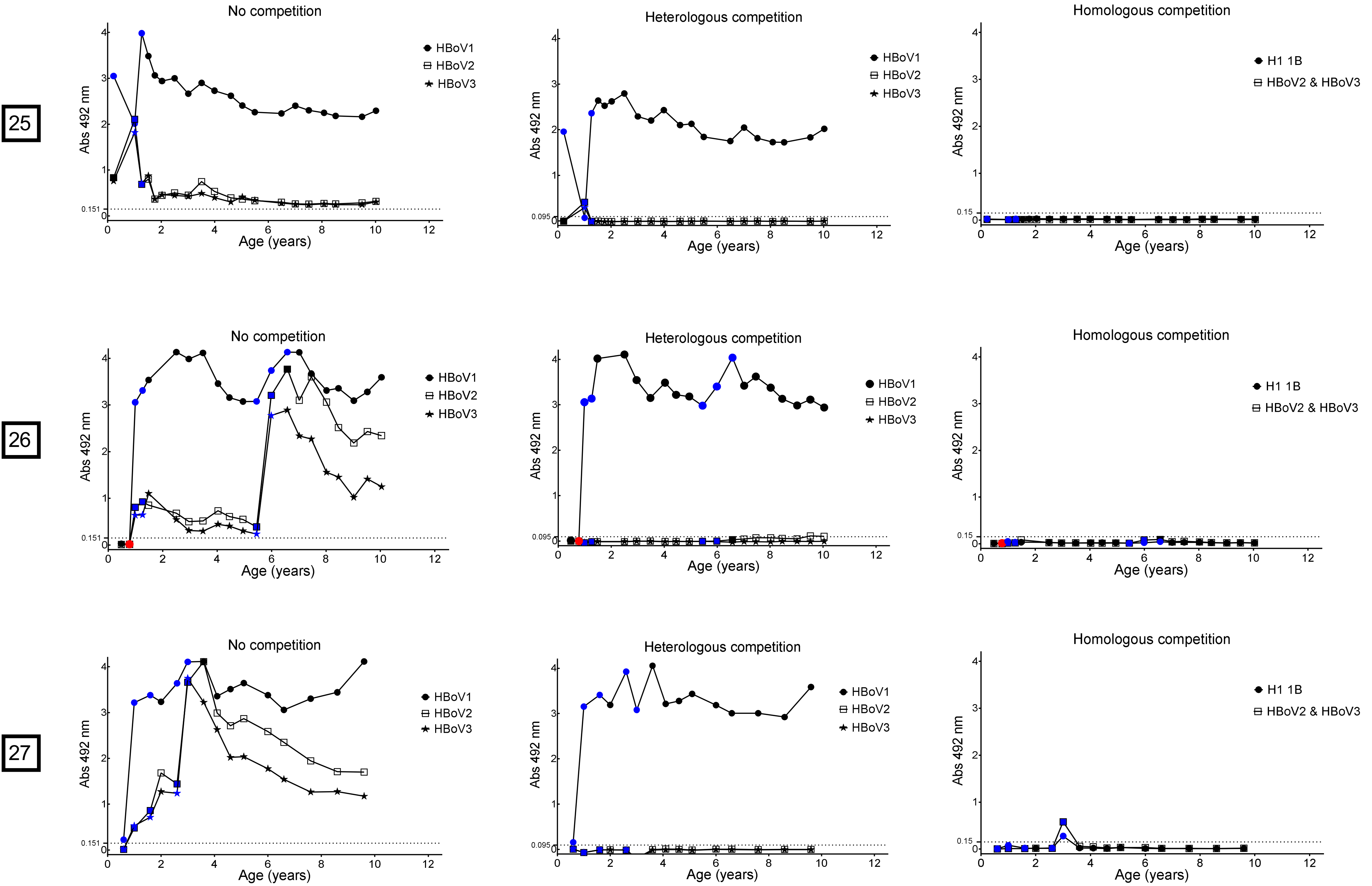
25
26
27
No competition
Heterologous competition
Homologous competition
HBoV1
HBoV2
HBoV3
H1 1B
HBoV2 & HBoV3
Abs 492 nm
Age (years)
0.151
0.095
0.15

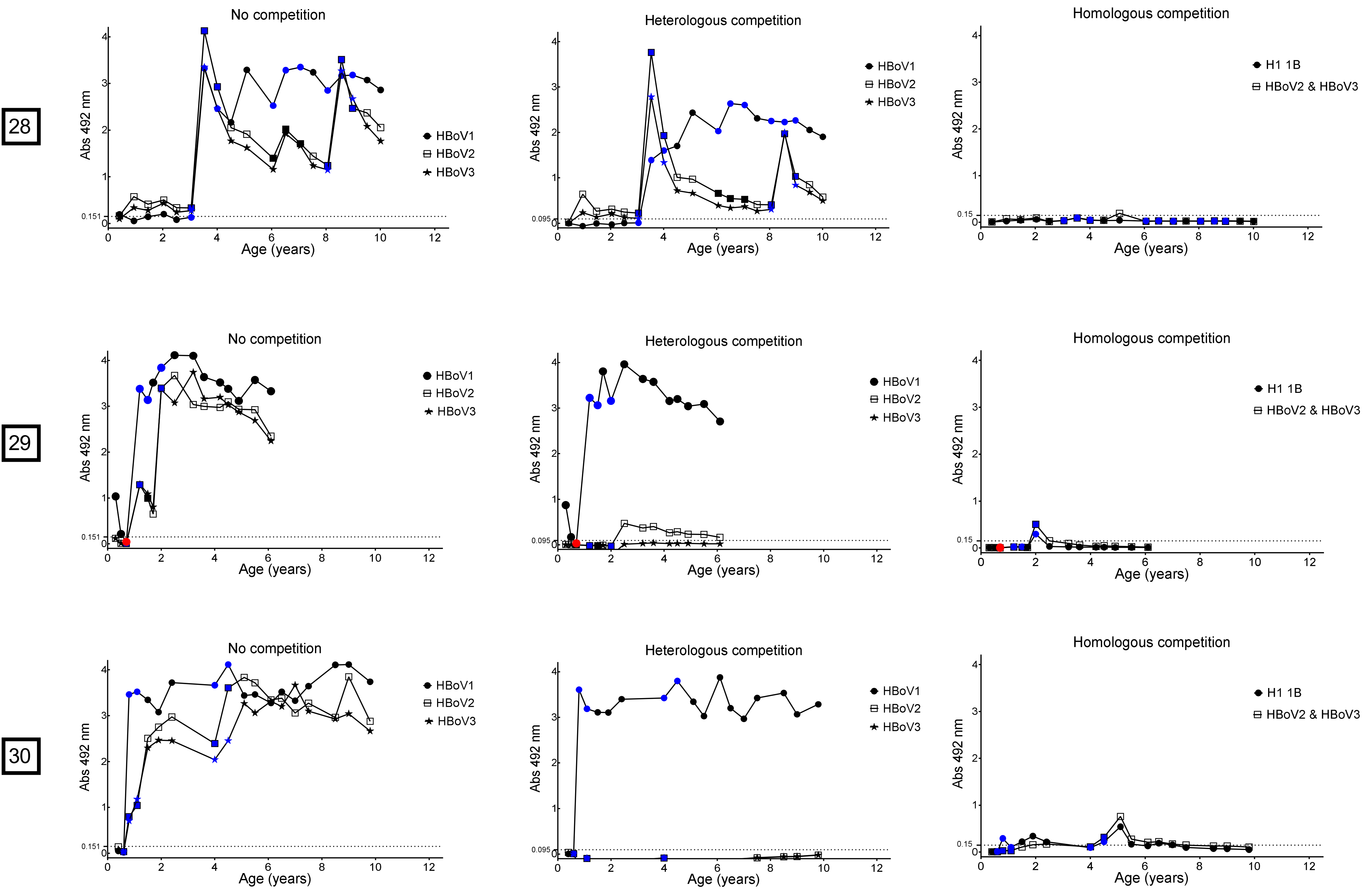
28
No competition
Heterologous competition
Homologous competition
29
No competition
Heterologous competition
Homologous competition
30
No competition
Heterologous competition
Homologous competition
Abs 492 nm
Age (years)
HBoV1
HBoV2
HBoV3
H1 1B
HBoV2 & HBoV3
0.151
0.095
0.15

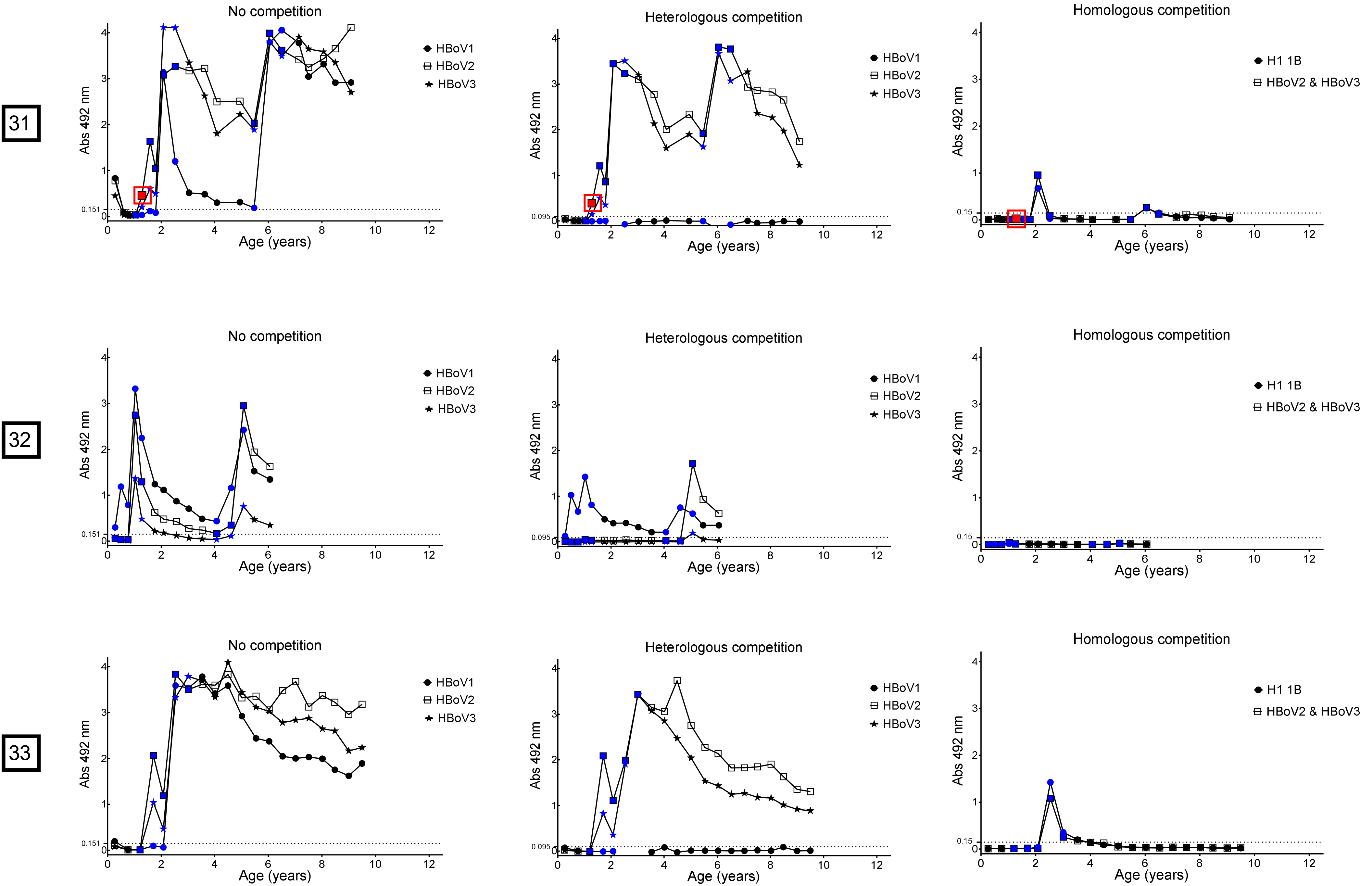
31
32
33
No competition
Heterologous competition
Homologous competition
Abs 492 nm
Age (years)
0.151
0.095
0.15
0
1
2
3
4
6
8
10
12
HBoV1
HBoV2
HBoV3
H1 1B
HBoV2 & HBoV3

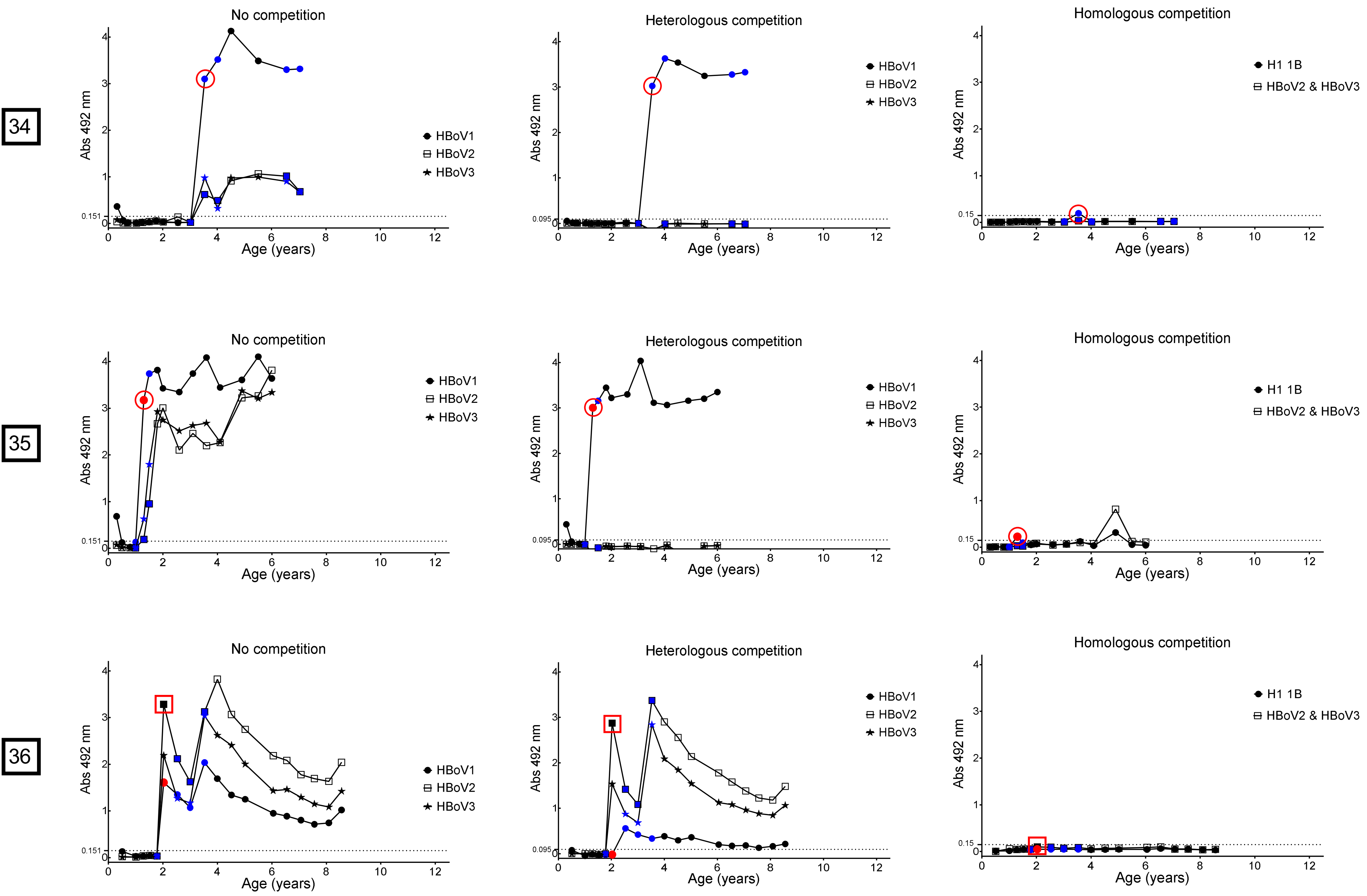
34
No competition
Abs 492 nm
Age (years)
0.151
HBoV1
HBoV2
HBoV3
Heterologous competition
0.095
Homologous competition
0.15
H1 1B
HBoV2 & HBoV3
35
No competition
Heterologous competition
Homologous competition
36
No competition
Heterologous competition
Homologous competition

37

38

39

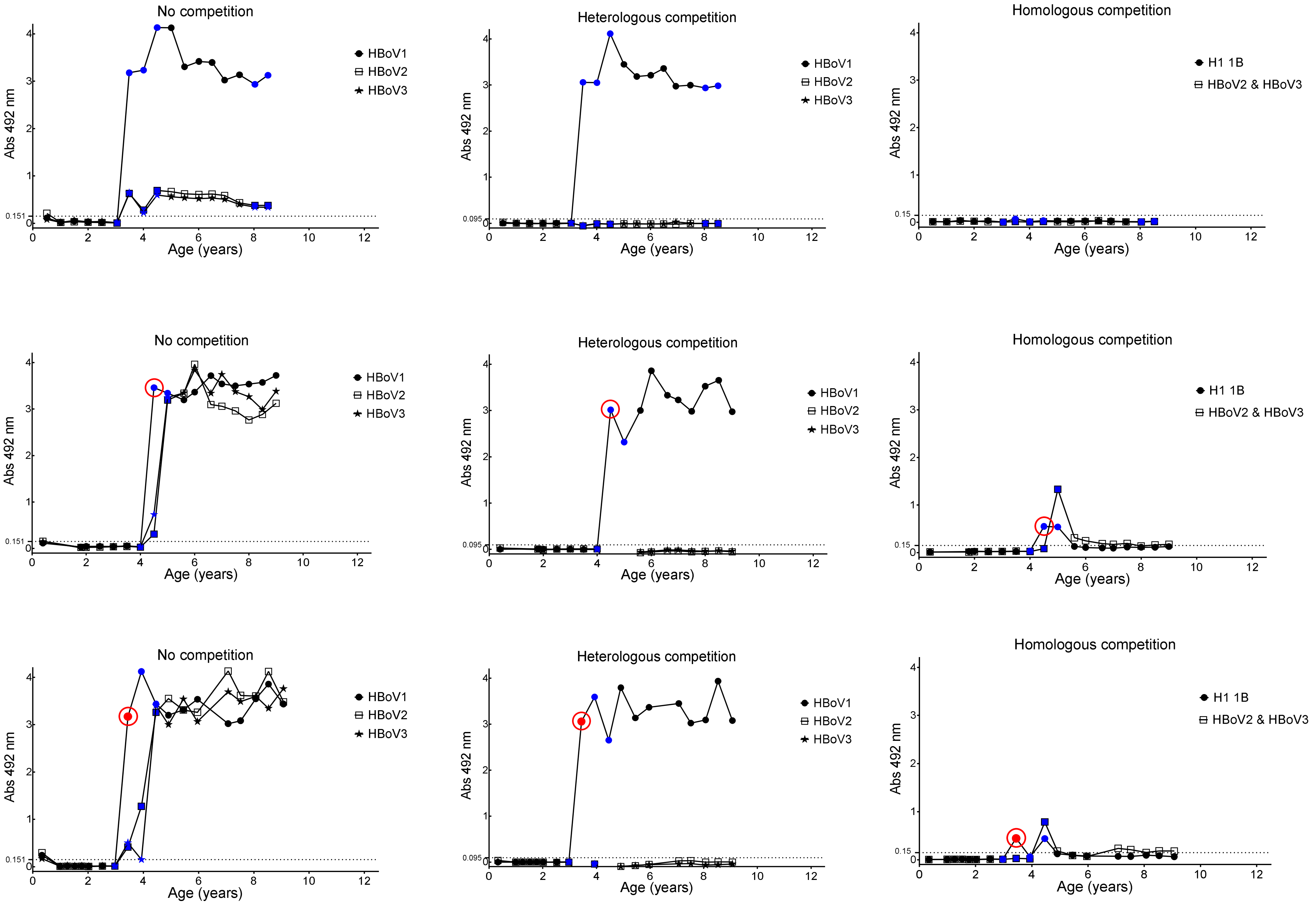

No competition
Heterologous competition
Homologous competition
Abs 492 nm
Age (years)
HBoV1
HBoV2
HBoV3
H1 1B
HBoV2 & HBoV3
0.151
0.095
0.15

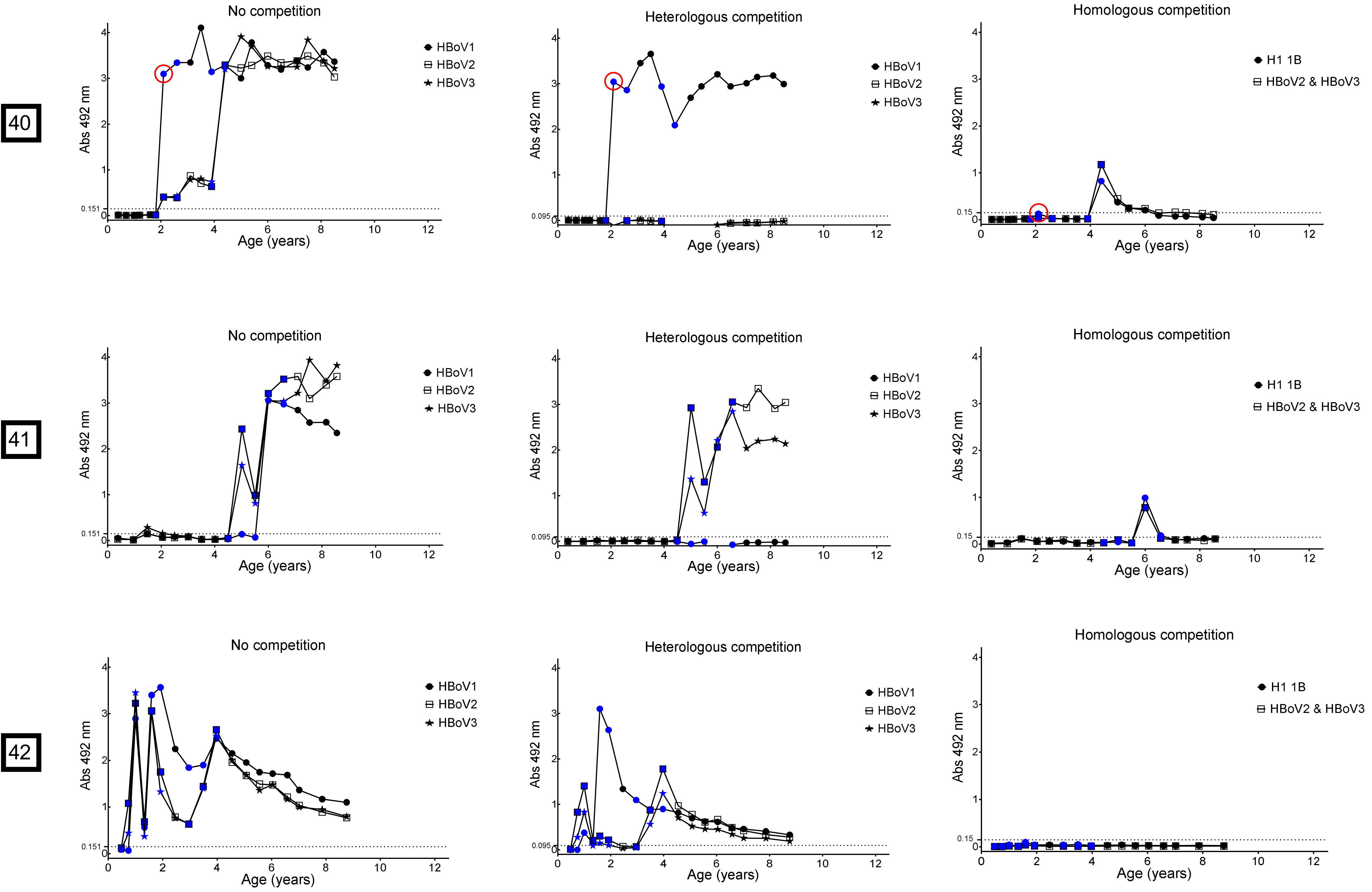
40
41
42
No competition
Heterologous competition
Homologous competition
HBoV1
HBoV2
HBoV3
H1 1B
HBoV2 & HBoV3
Abs 492 nm
Age (years)
0.151
0.095
0.15

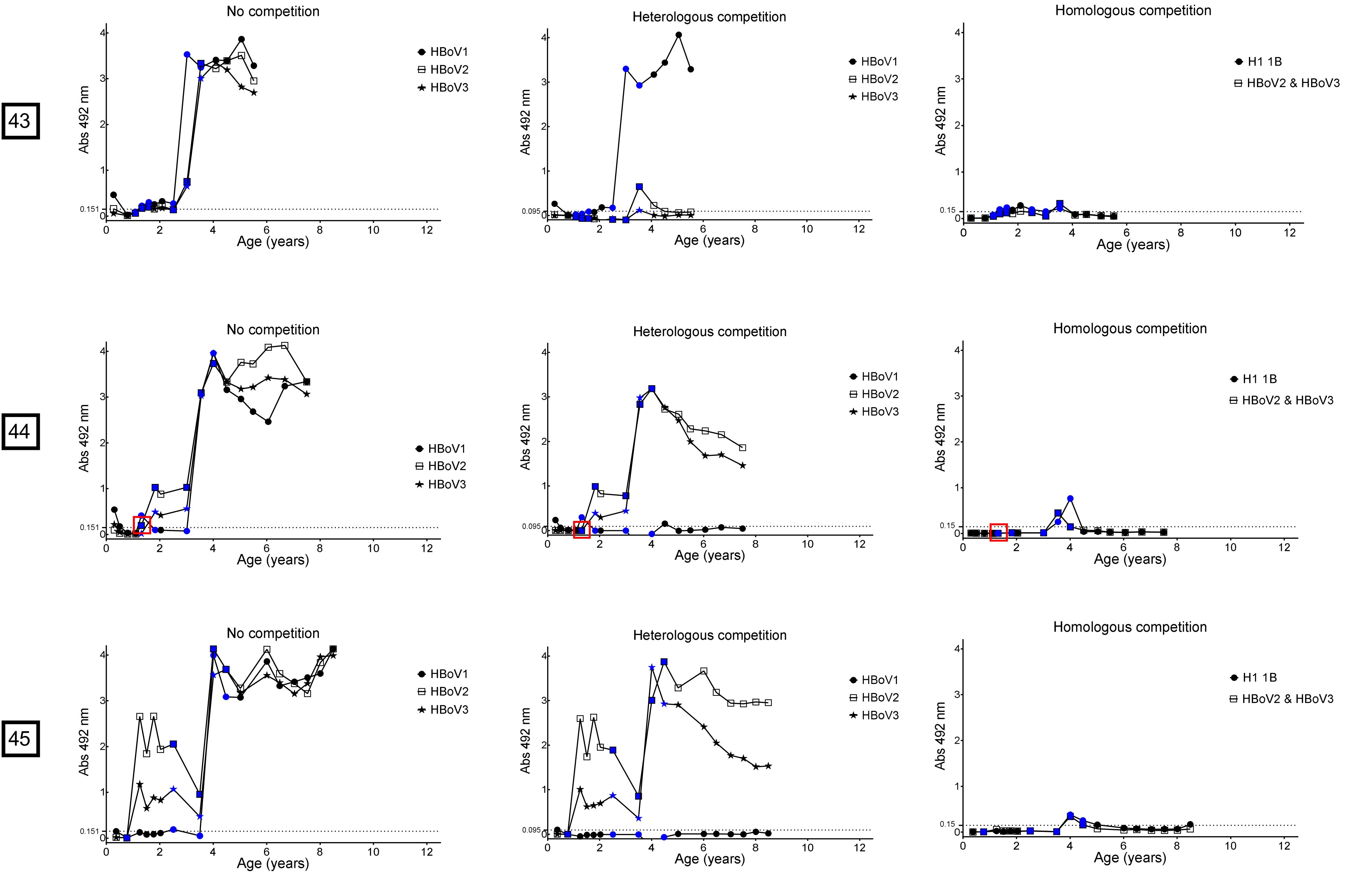
43
44
45
No competition
Heterologous competition
Homologous competition
Abs 492 nm
Age (years)
HBoV1
HBoV2
HBoV3
H1 1B
HBoV2 & HBoV3
0.151
0.095
0.15

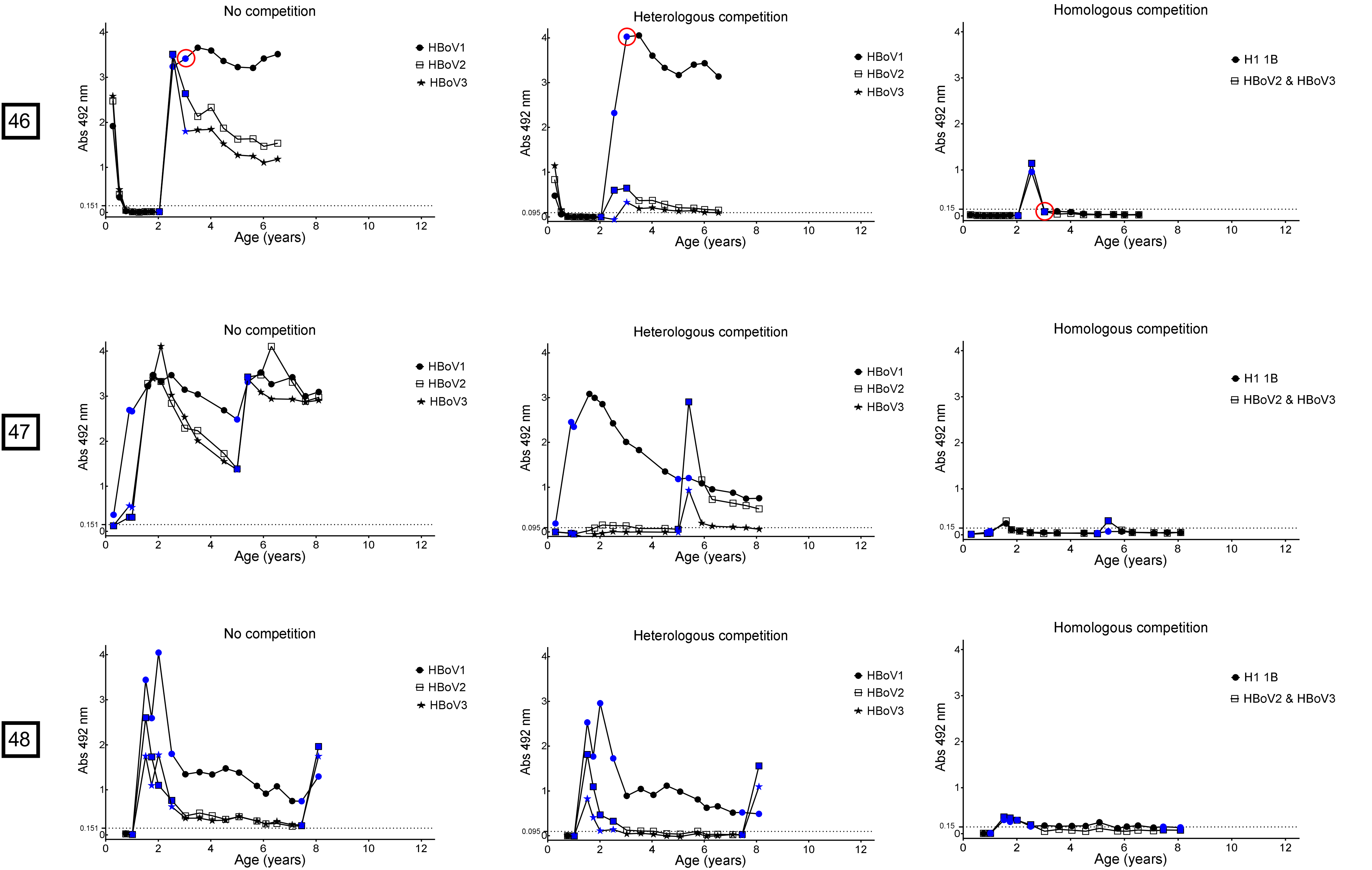

46
No competition
Heterologous competition
Homologous competition
HBoV1
HBoV2
HBoV3
H1 1B
HBoV2 & HBoV3
Abs 492 nm
Age (years)
0.151
0.095
0.15
47
No competition
Heterologous competition
Homologous competition
HBoV1
HBoV2
HBoV3
H1 1B
HBoV2 & HBoV3
Abs 492 nm
Age (years)
0.151
0.095
0.15
48
No competition
Heterologous competition
Homologous competition
HBoV1
HBoV2
HBoV3
H1 1B
HBoV2 & HBoV3
Abs 492 nm
Age (years)
0.151
0.095
0.15

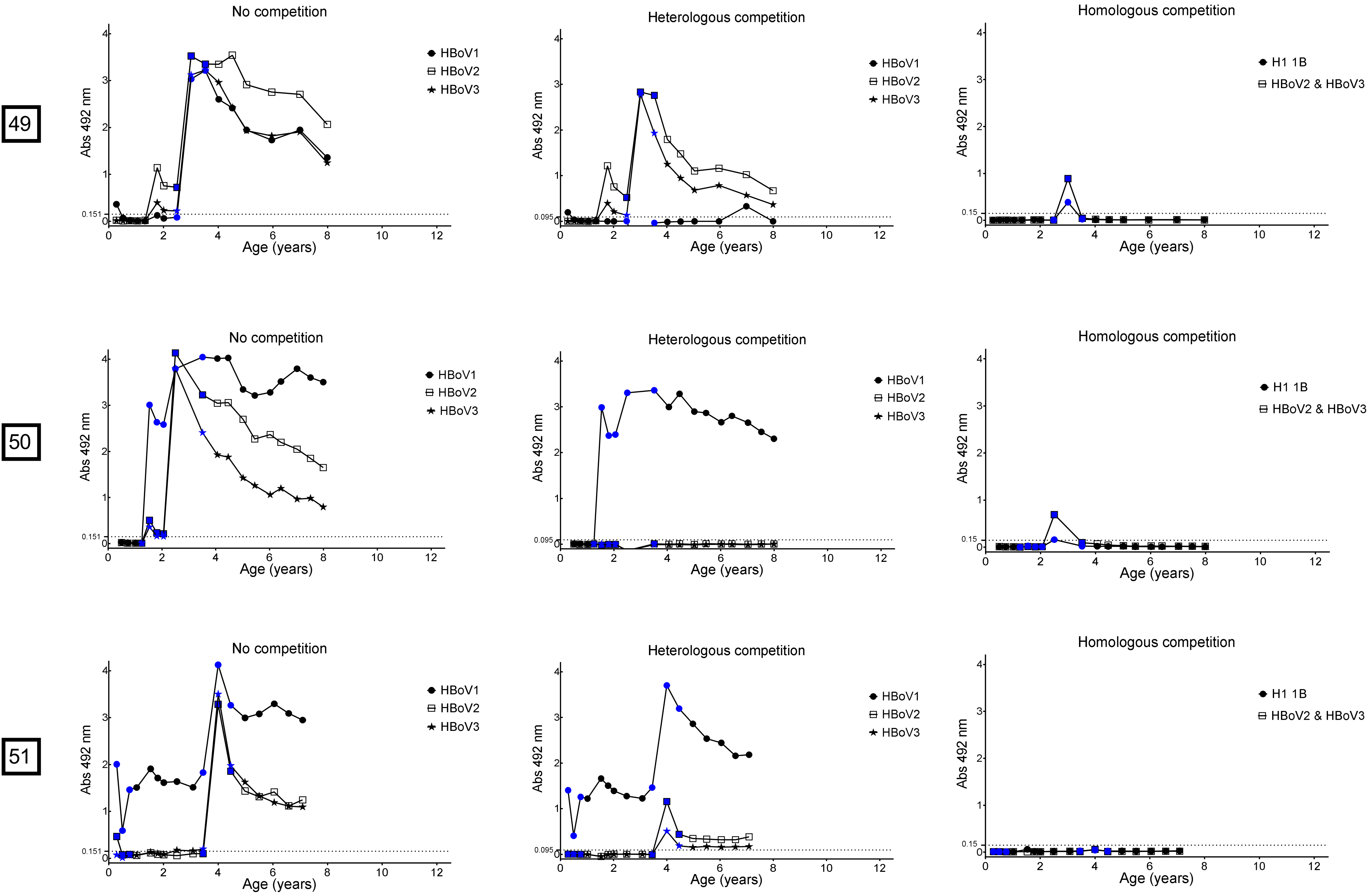
49
50
51
No competition
Heterologous competition
Homologous competition
HBoV1
HBoV2
HBoV3
H1 1B
HBoV2 & HBoV3
Abs 492 nm
Age (years)
0.151
0.095
0.15

52

53

54

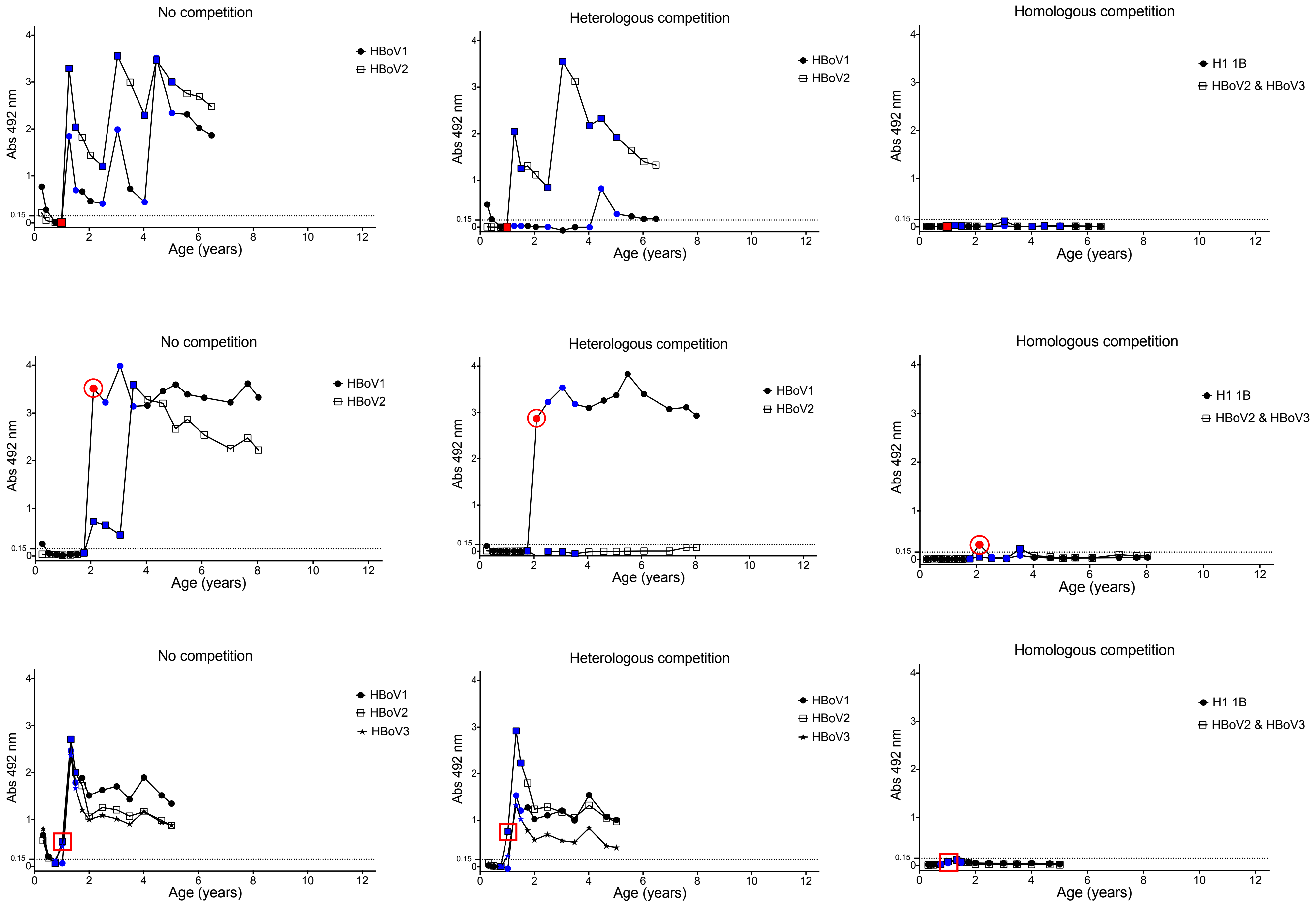
No competition
Heterologous competition
Homologous competition
HBoV1
HBoV2
HBoV3
H1 1B
HBoV2 & HBoV3
Abs 492 nm
Age (years)
0.15

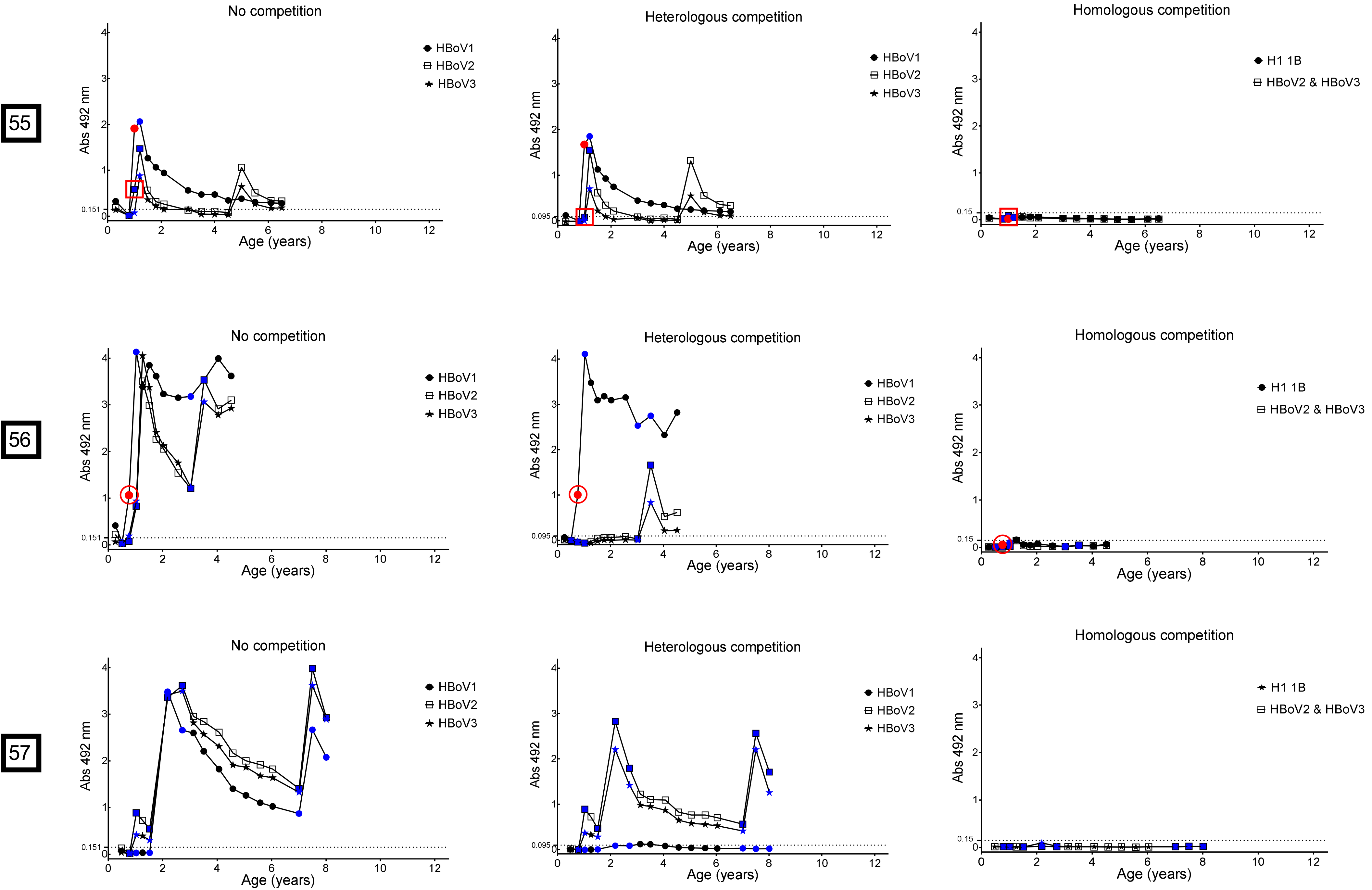
55
56
57
No competition
Heterologous competition
Homologous competition
HBoV1
HBoV2
HBoV3
H1 1B
HBoV2 & HBoV3
Abs 492 nm
Age (years)
0.151
0.095
0.15
0
1
2
3
4
6
8
10
12

58

59

60

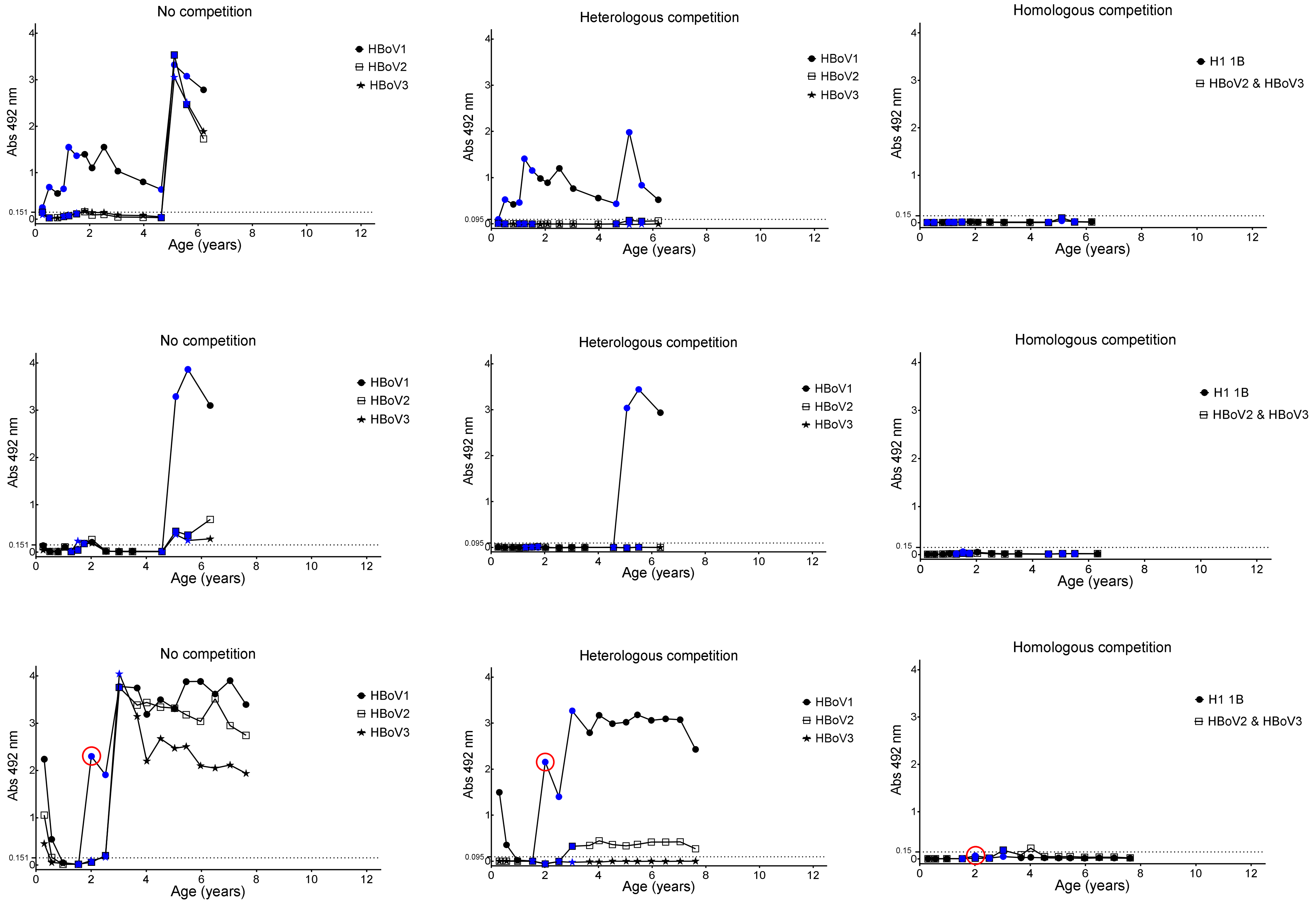
No competition
Heterologous competition
Homologous competition
Abs 492 nm
Age (years)
0.151
0.095
0.15
HBoV1
HBoV2
HBoV3
H1 1B
HBoV2 & HBoV3

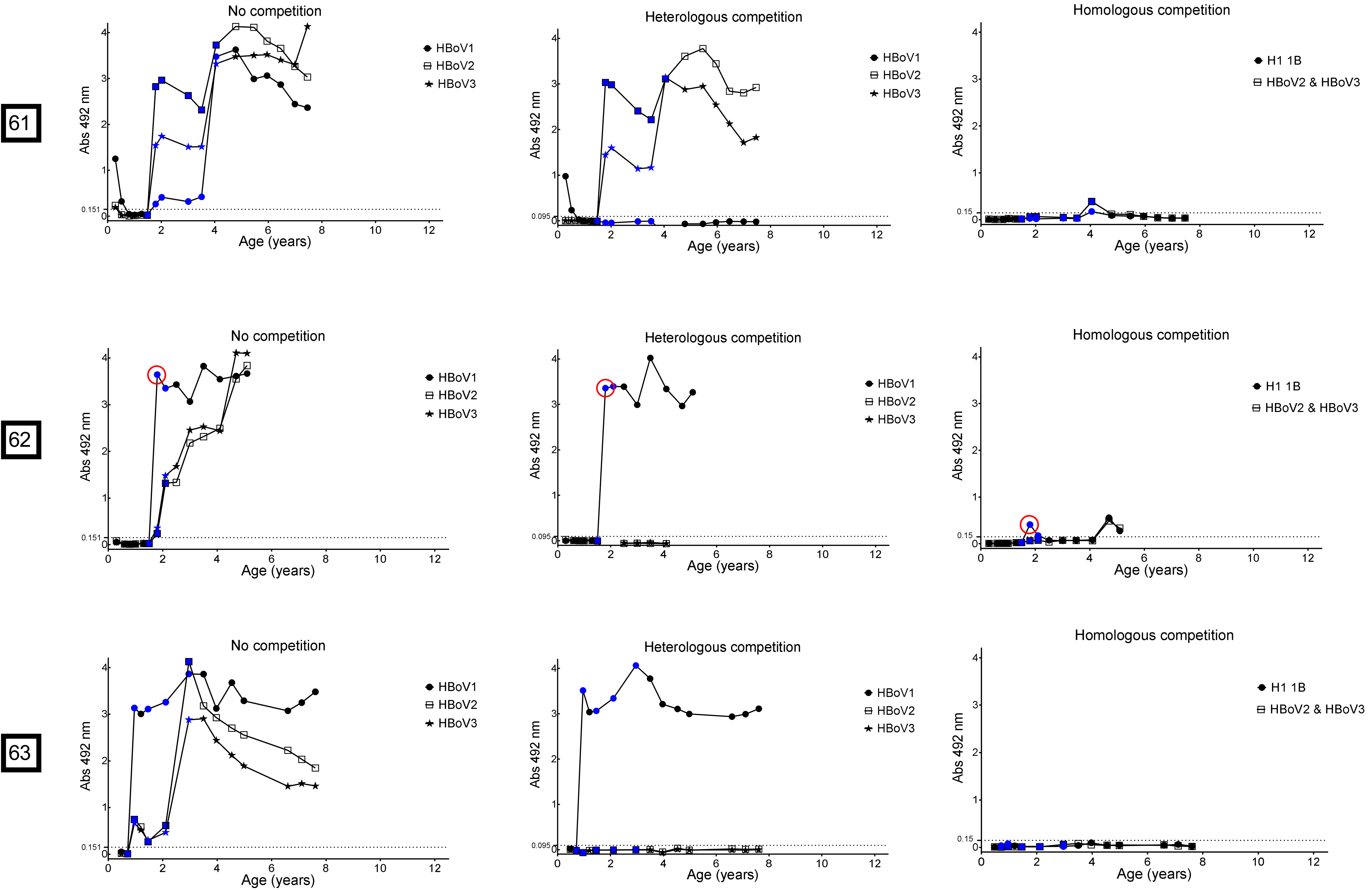
61
62
63
No competition
Heterologous competition
Homologous competition
HBoV1
HBoV2
HBoV3
H1 1B
HBoV2 & HBoV3
Abs 492 nm
Age (years)
0.151
0.095
0.15
0
1
2
3
4
6
8
10
12

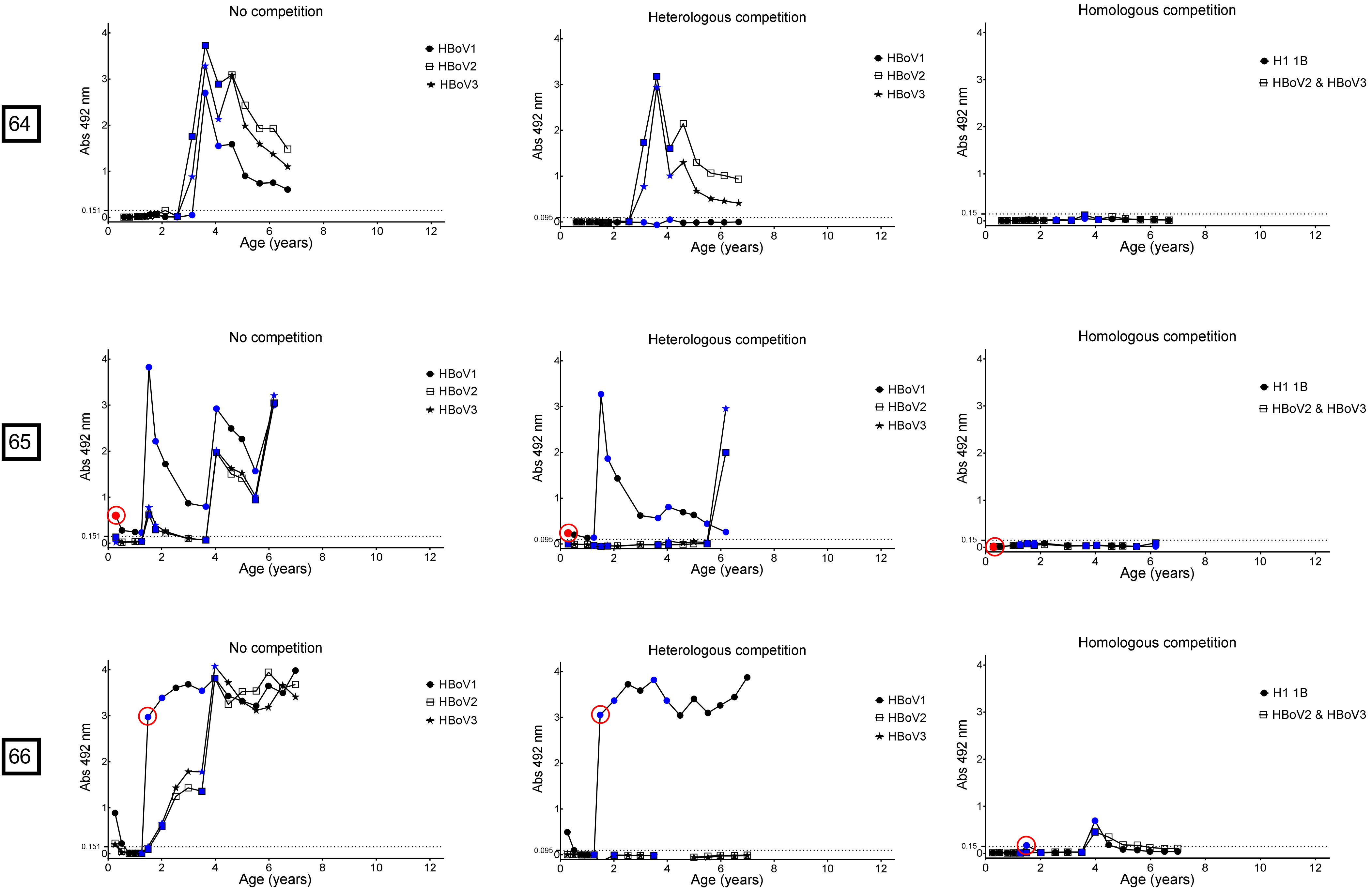

64
65
66
No competition
Heterologous competition
Homologous competition
Abs 492 nm
Age (years)
0.151
0.095
0.15
HBoV1
HBoV2
HBoV3
H1 1B
HBoV2 & HBoV3

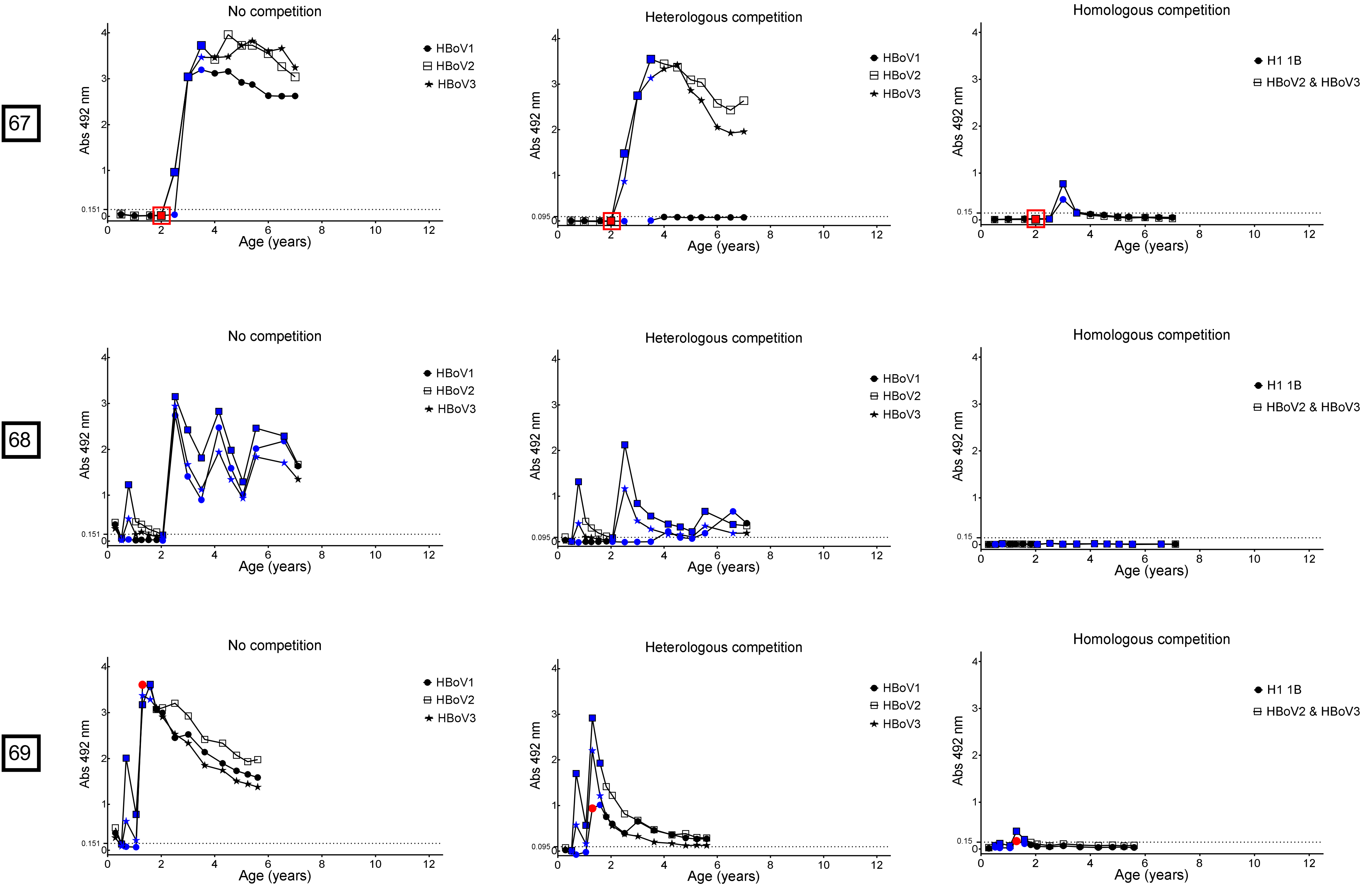
67
68
69
No competition
Heterologous competition
Homologous competition
Abs 492 nm
Age (years)
0.151
0.095
0.15
HBoV1
HBoV2
HBoV3
H1 1B
HBoV2 & HBoV3

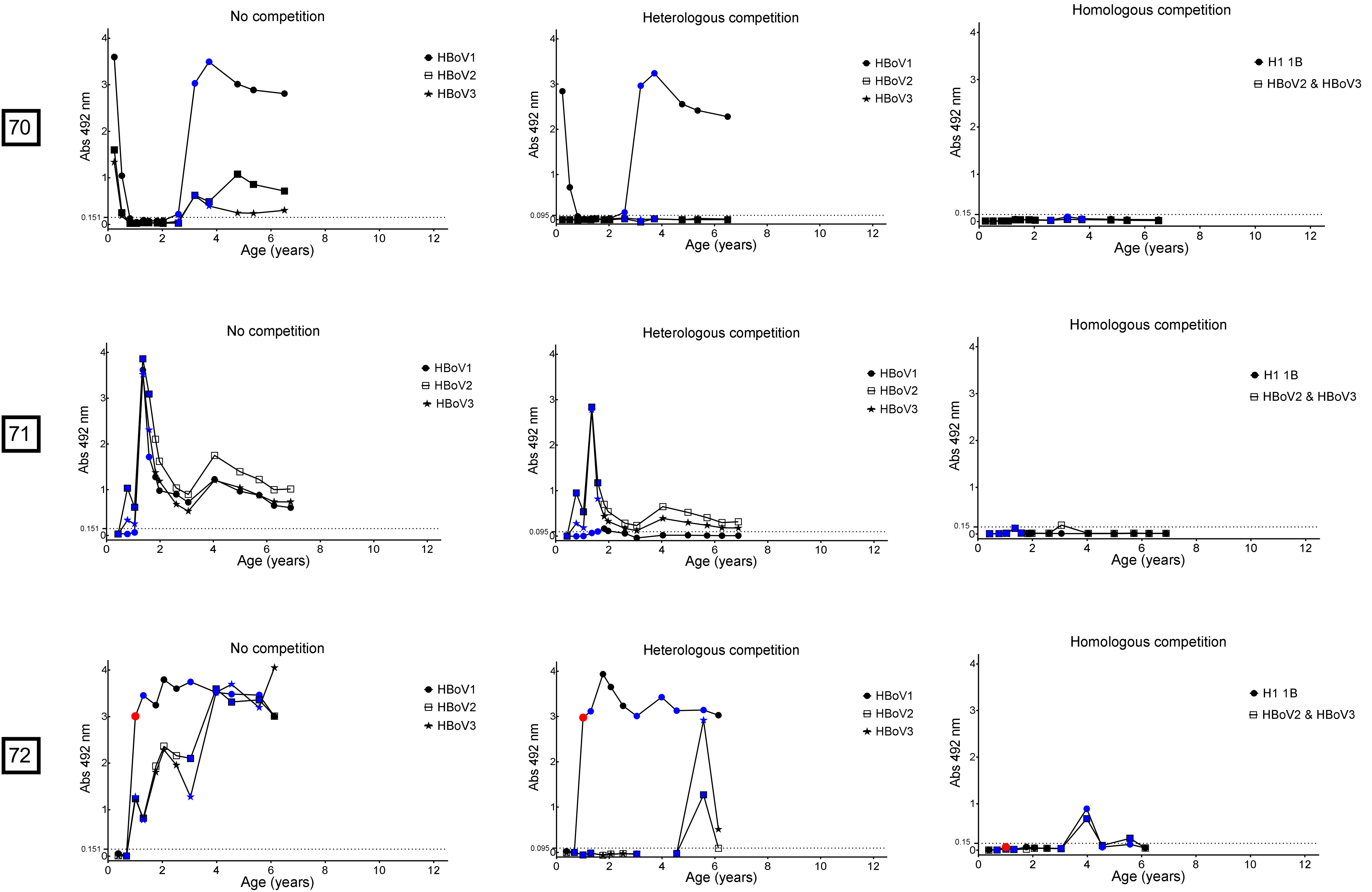
70
71
72
No competition
Heterologous competition
Homologous competition
Abs 492 nm
Age (years)
HBoV1
HBoV2
HBoV3
H1 1B
HBoV2 & HBoV3
0.151
0.095
0.15
0
1
2
3
4
6
8
10
12

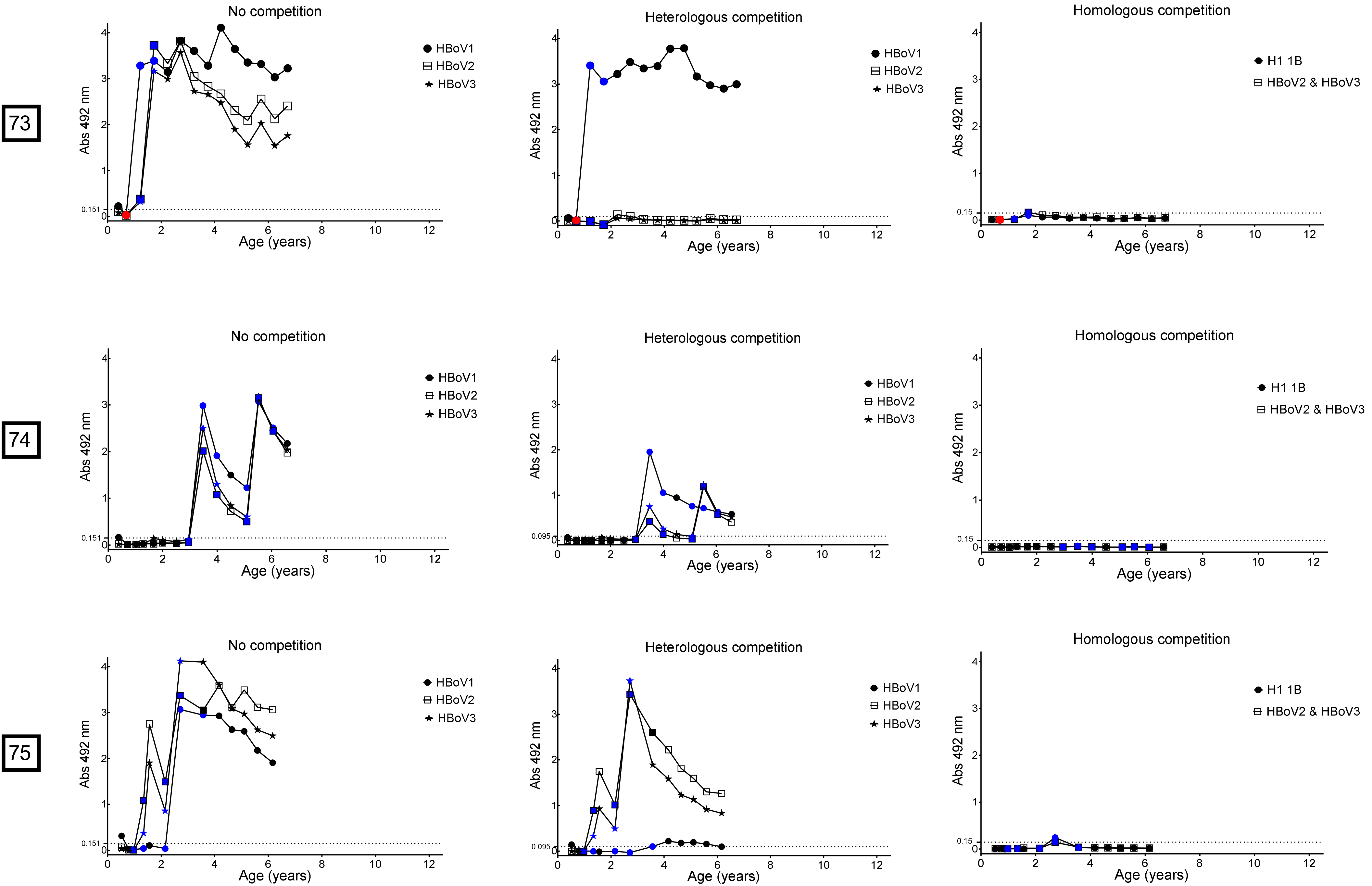
73
74
75
No competition
Heterologous competition
Homologous competition
Abs 492 nm
Age (years)
HBoV1
HBoV2
HBoV3
H1 1B
HBoV2 & HBoV3
0.151
0.095
0.15
0
1
2
3
4
6
8
10
12

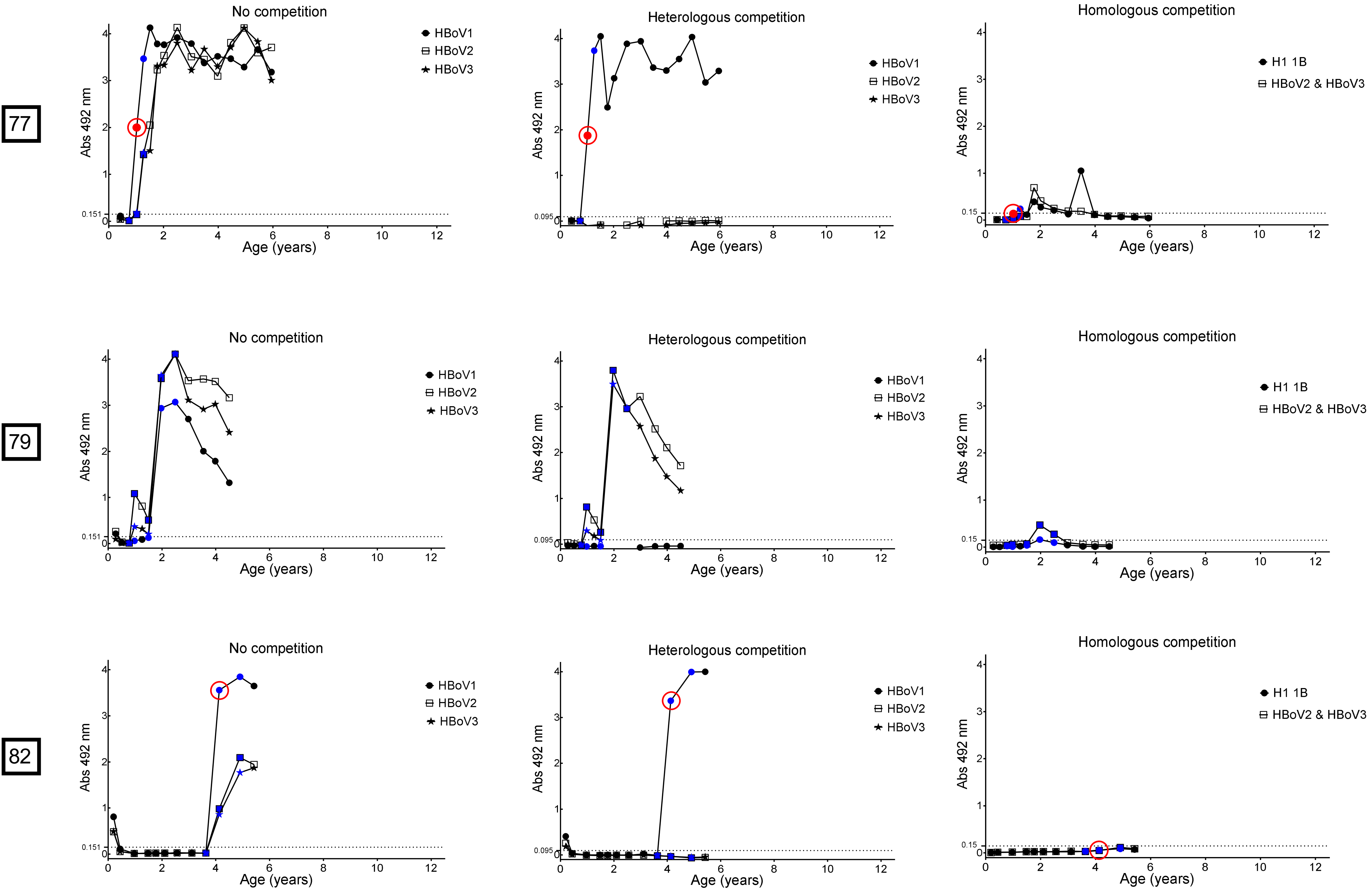

77
No competition
Heterologous competition
Homologous competition
HBoV1
HBoV2
HBoV3
H1 1B
HBoV2 & HBoV3
Abs 492 nm
Age (years)
0.151
0.095
0.15
79
No competition
Heterologous competition
Homologous competition
82
No competition
Heterologous competition
Homologous competition

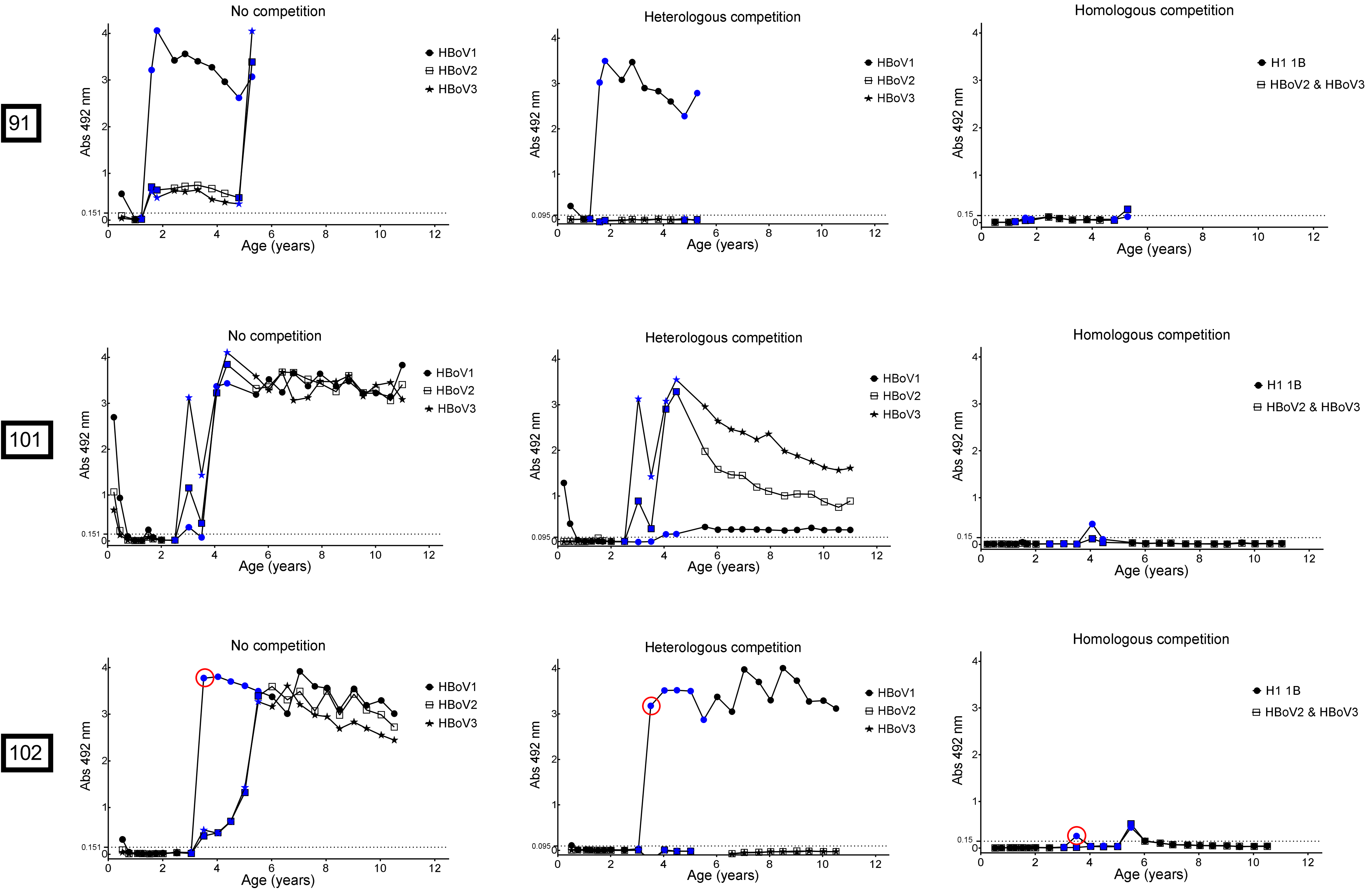

91
No competition
Heterologous competition
Homologous competition
HBoV1
HBoV2
HBoV3
H1 1B
HBoV2 & HBoV3
Abs 492 nm
Age (years)
0.151
0.095
0.15
101
No competition
Heterologous competition
Homologous competition
HBoV1
HBoV2
HBoV3
H1 1B
HBoV2 & HBoV3
Abs 492 nm
Age (years)
0.151
0.095
0.15
102
No competition
Heterologous competition
Homologous competition
HBoV1
HBoV2
HBoV3
H1 1B
HBoV2 & HBoV3
Abs 492 nm
Age (years)
0.151
0.095
0.15

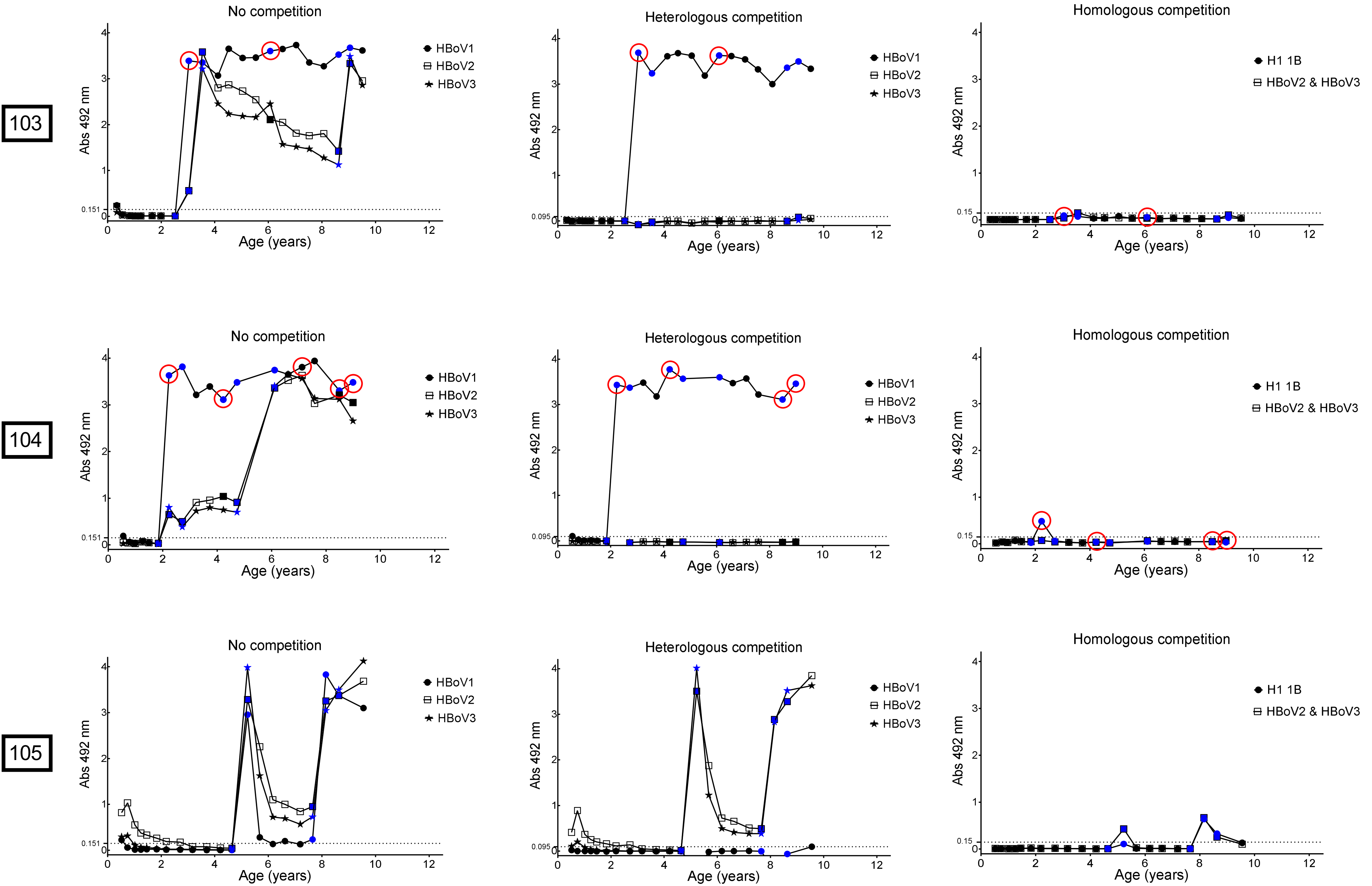
103
104
105
No competition
Heterologous competition
Homologous competition
Abs 492 nm
Age (years)
HBoV1
HBoV2
HBoV3
H1 1B
HBoV2 & HBoV3
0.151
0.095
0.15

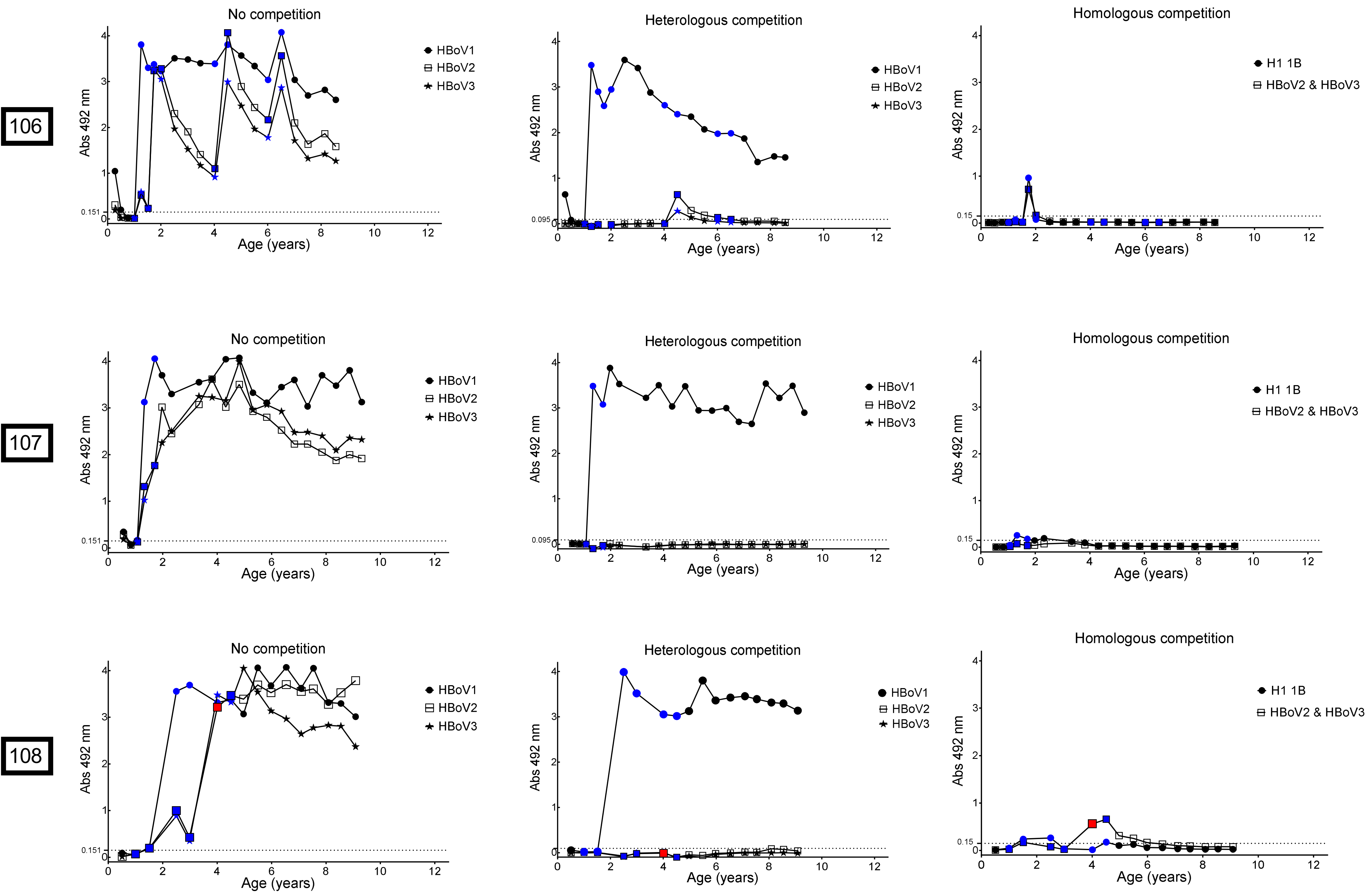

106
107
108
No competition
Heterologous competition
Homologous competition
Abs 492 nm
Age (years)
0.151
0.095
0.15
HBoV1
HBoV2
HBoV3
H1 1B
HBoV2 & HBoV3

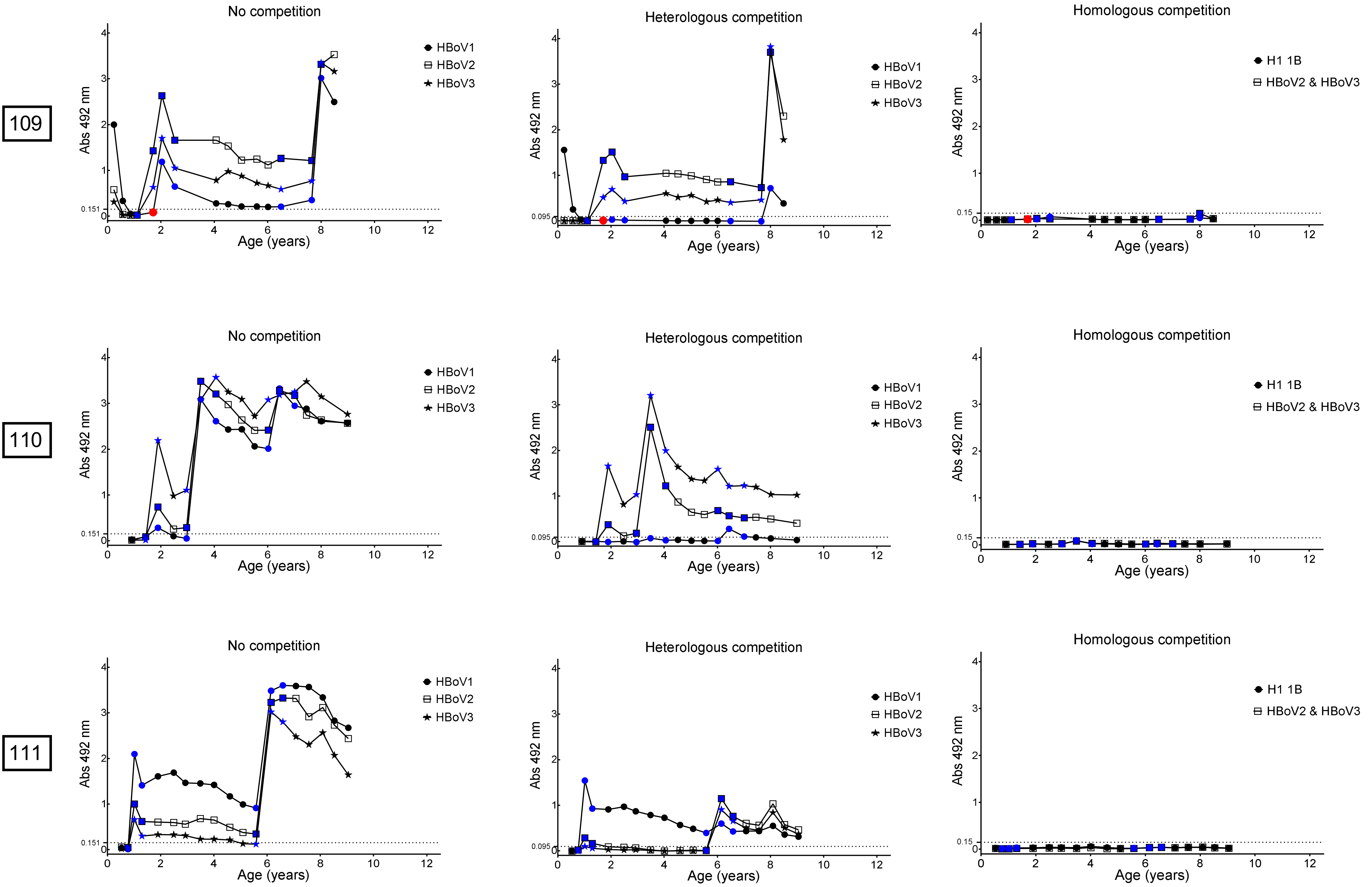
109
110
111
No competition
Heterologous competition
Homologous competition
HBoV1
HBoV2
HBoV3
H1 1B
HBoV2 & HBoV3
Abs 492 nm
Age (years)
0.151
0.095
0.15

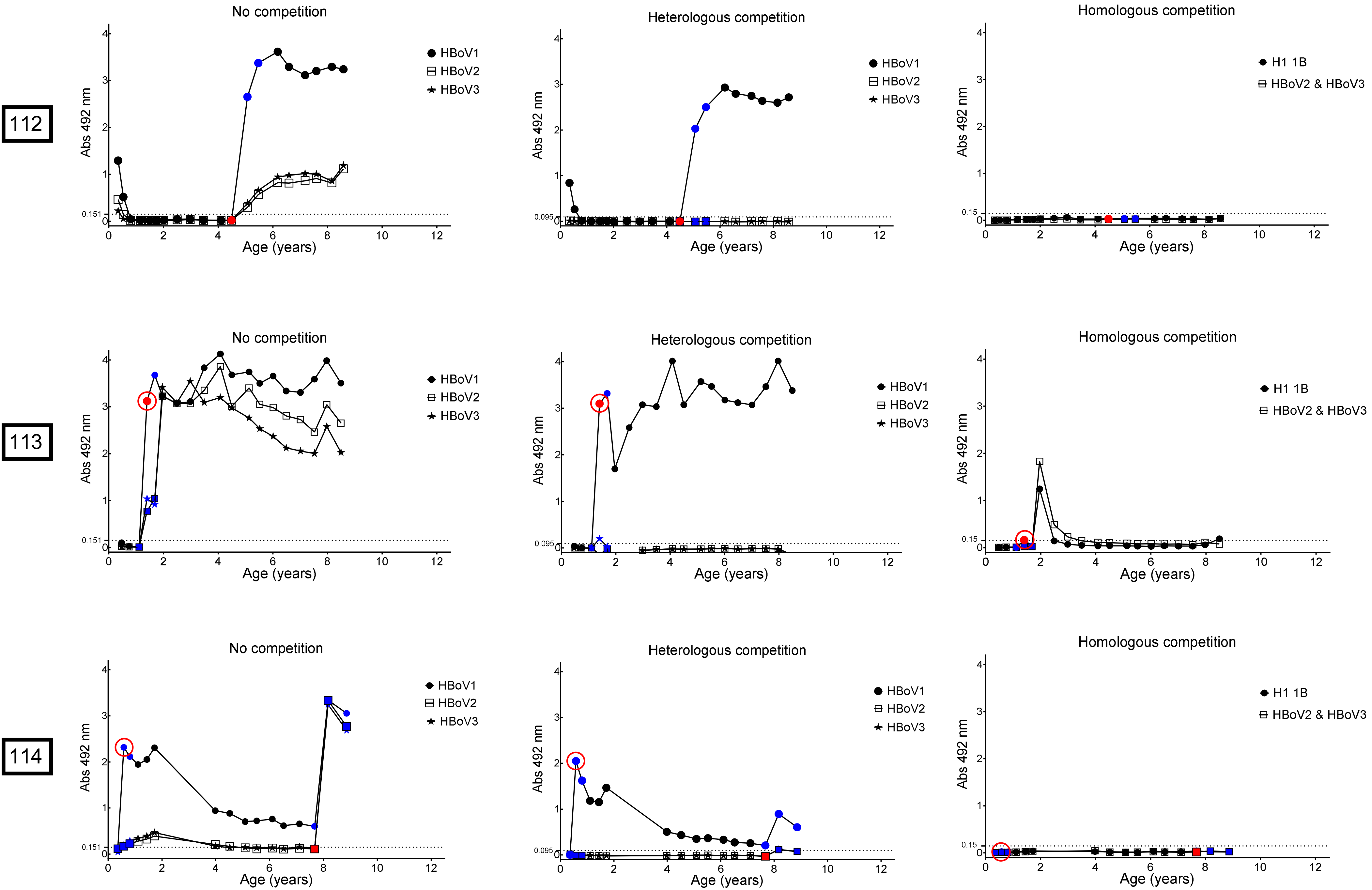

112
113
114
No competition
Heterologous competition
Homologous competition
Abs 492 nm
Age (years)
HBoV1
HBoV2
HBoV3
H1 1B
HBoV2 & HBoV3
0.151
0.095
0.15
0
1
2
3
4
0
2
4
6
8
10
12

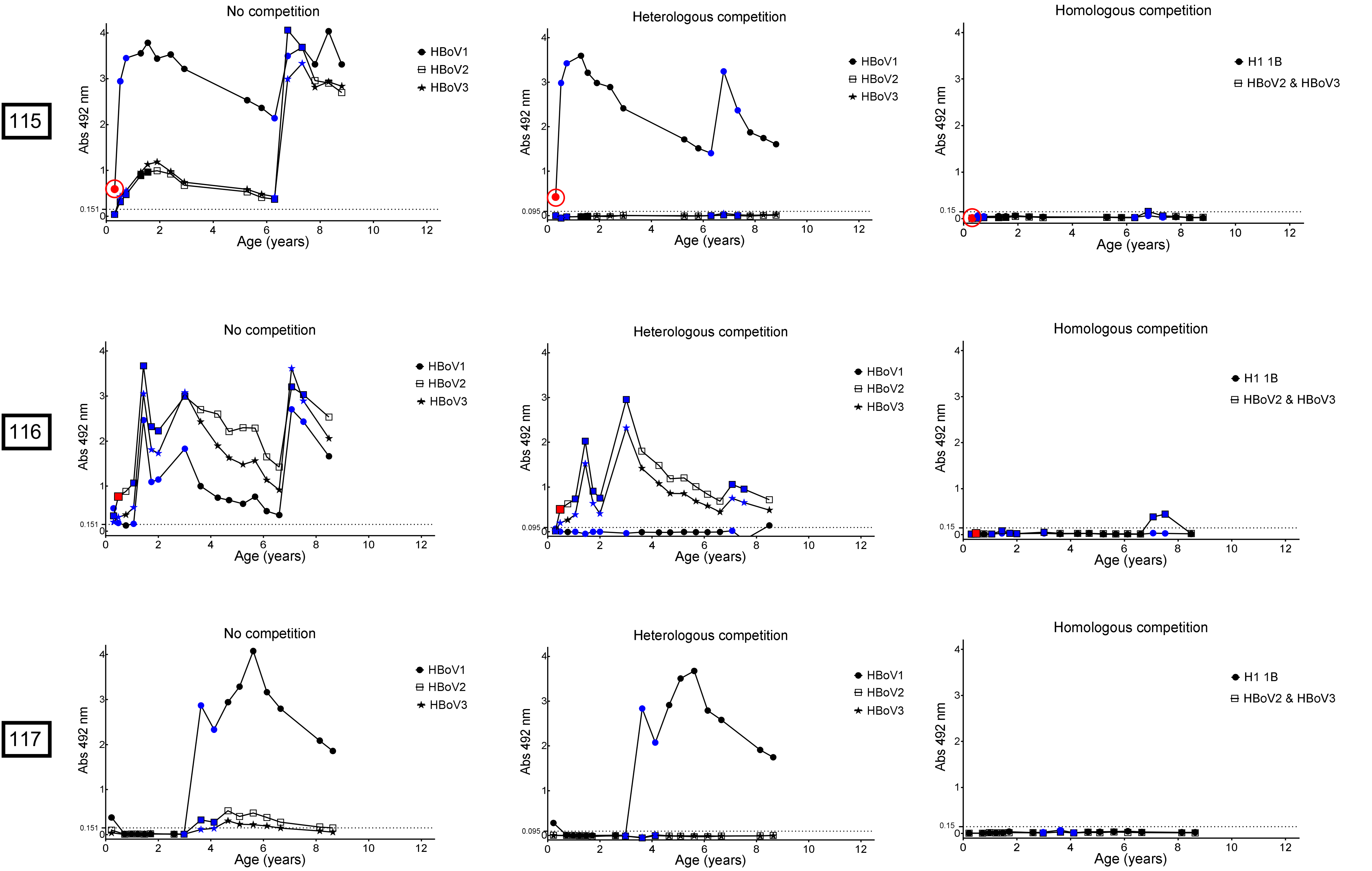
115
No competition
Abs 492 nm
Age (years)
0.151
HBoV1
HBoV2
HBoV3
Heterologous competition
0.095
Homologous competition
0.15
H1 1B
HBoV2 & HBoV3
116
117
0 2 4 6 8 10 12
0 1 2 3 4

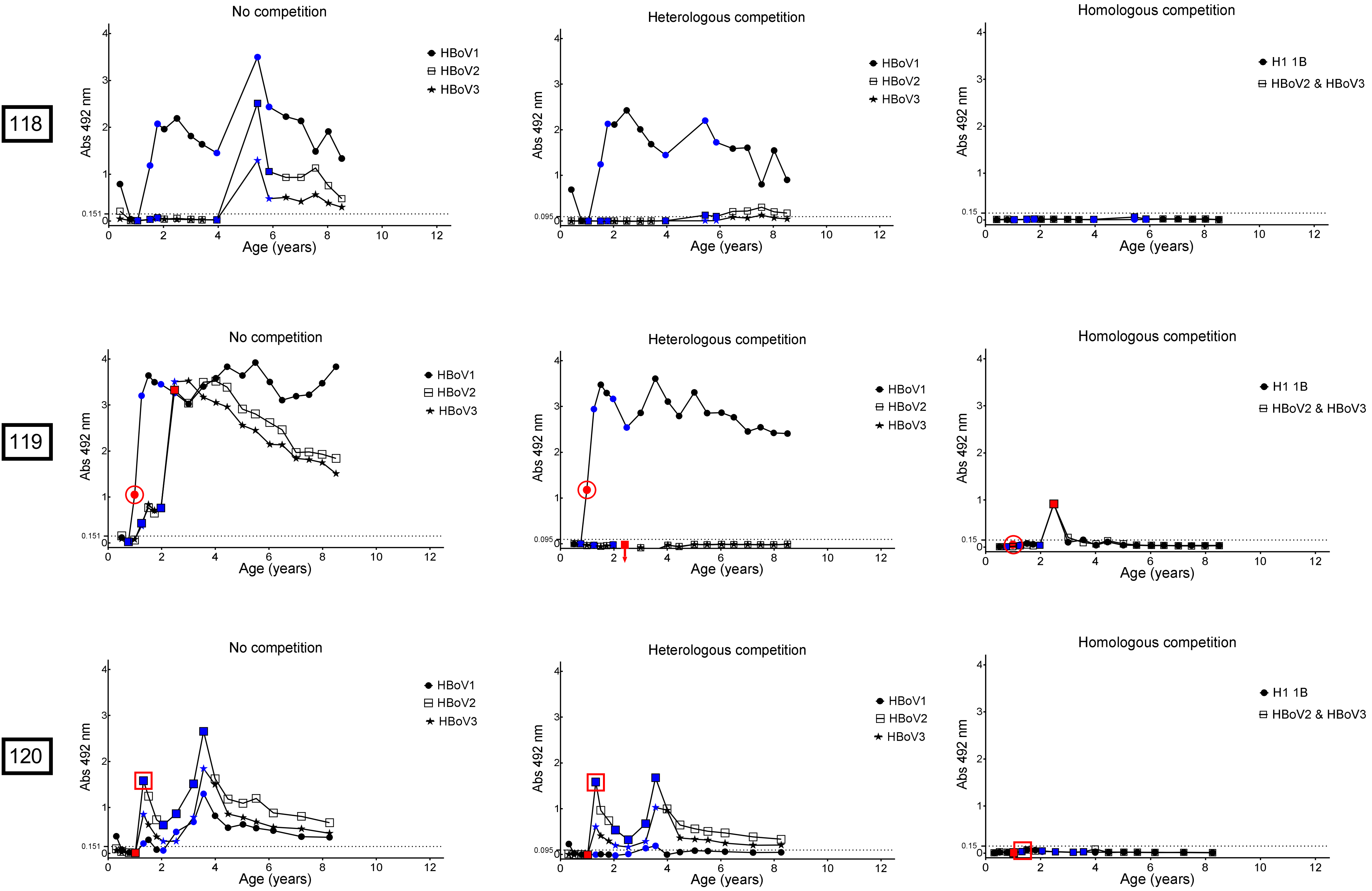
118
119
120
No competition
Heterologous competition
Homologous competition
Abs 492 nm
Age (years)
0.151
0.095
0.15
HBoV1
HBoV2
HBoV3
H1 1B
HBoV2 & HBoV3

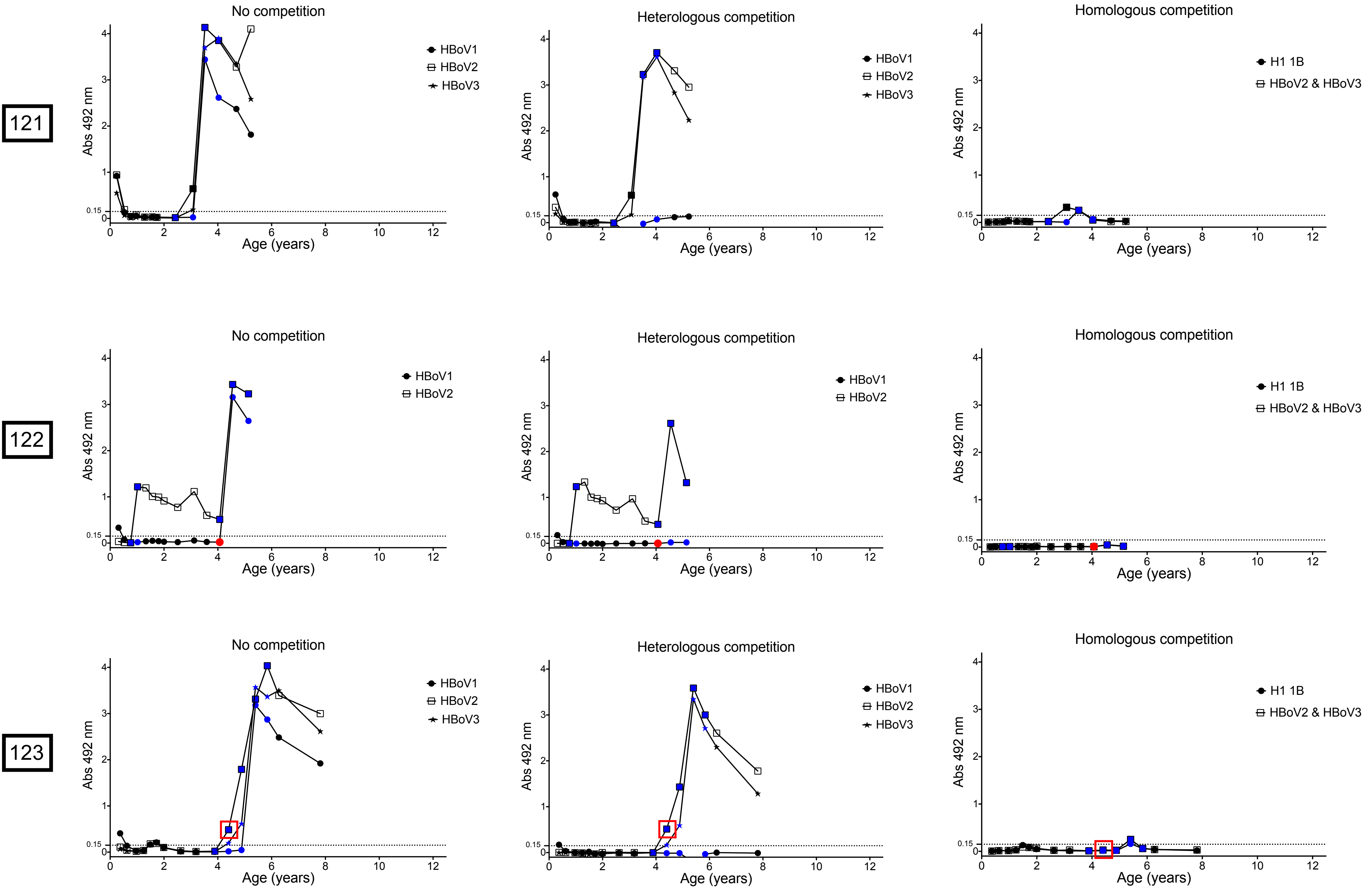

121
No competition
HBoV1
HBoV2
HBoV3
Heterologous competition
HBoV1
HBoV2
HBoV3
Homologous competition
H1 1B
HBoV2 & HBoV3
122
No competition
HBoV1
HBoV2
Heterologous competition
HBoV1
HBoV2
Homologous competition
H1 1B
HBoV2 & HBoV3
123
No competition
HBoV1
HBoV2
HBoV3
Heterologous competition
HBoV1
HBoV2
HBoV3
Homologous competition
H1 1B
HBoV2 & HBoV3
Abs 492 nm
Age (years)
0.15

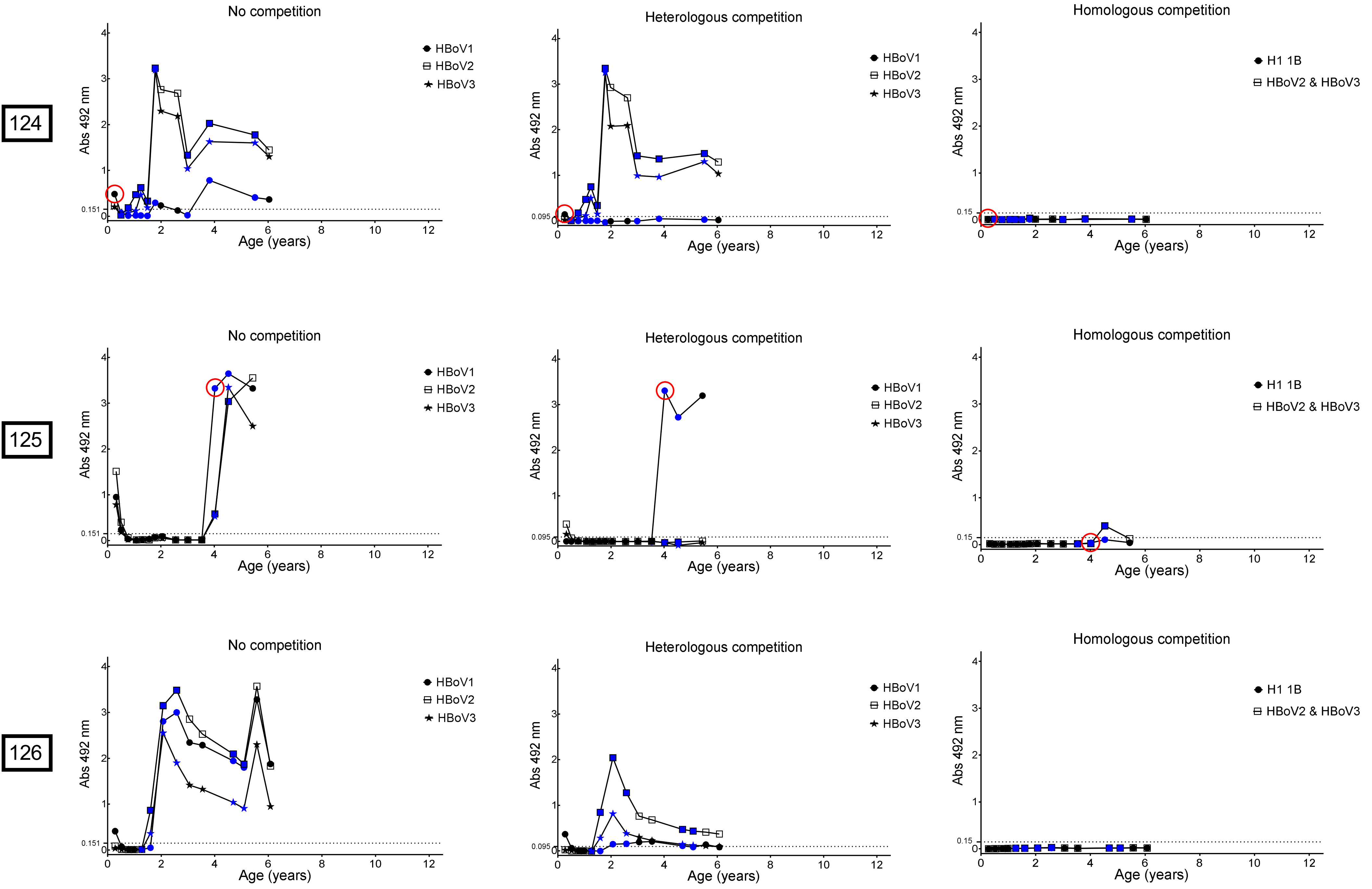
124
125
126
No competition
Heterologous competition
Homologous competition
HBoV1
HBoV2
HBoV3
H1 1B
HBoV2 & HBoV3
Abs 492 nm
Age (years)
0.151
0.095
0.15
0
1
2
3
4
6
8
10
12

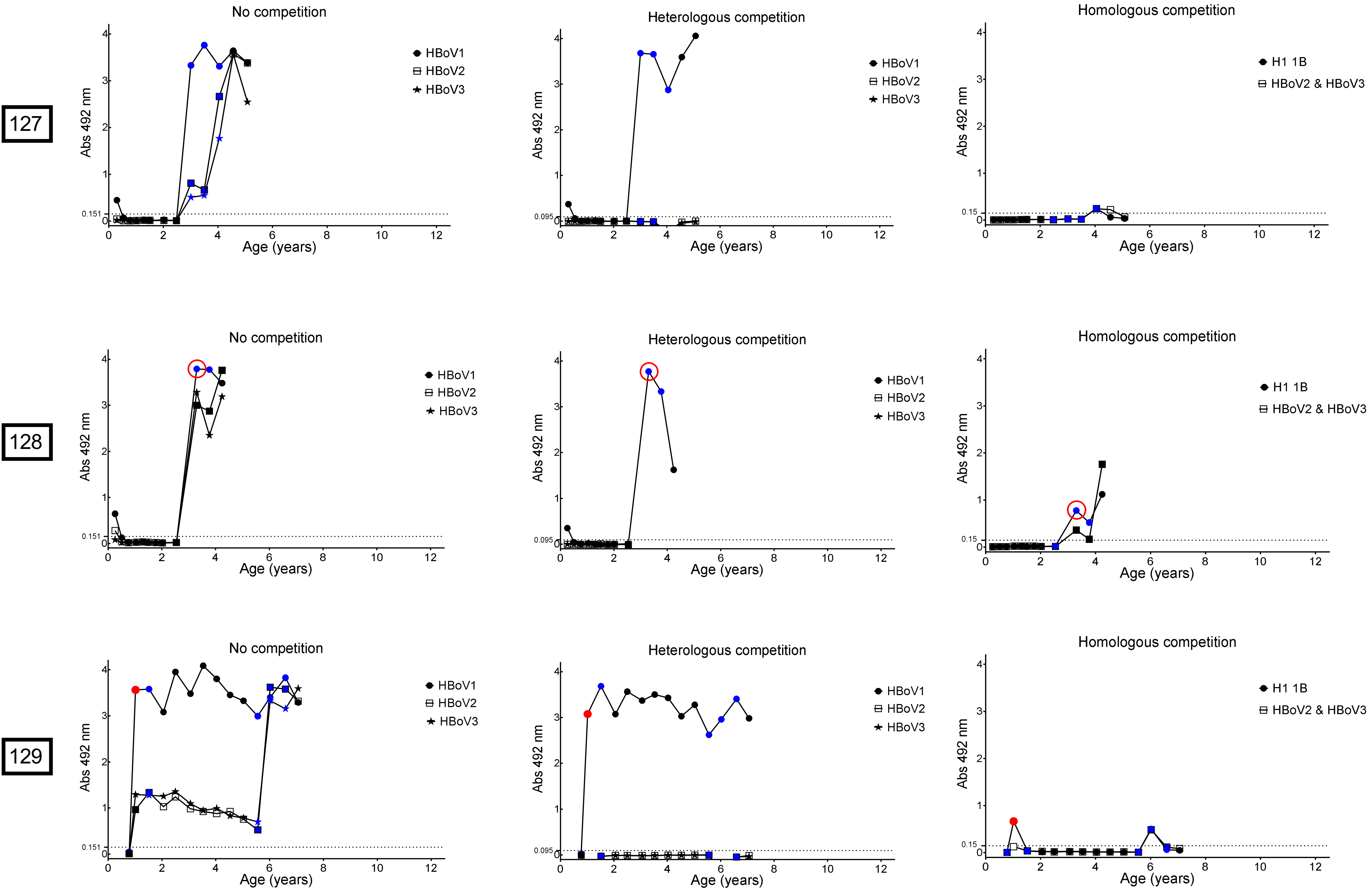

127
No competition
Abs 492 nm
Age (years)
0.151
HBoV1
HBoV2
HBoV3
Heterologous competition
0.095
Homologous competition
0.15
H1 1B
HBoV2 & HBoV3
128
No competition
Heterologous competition
Homologous competition
129
No competition
Heterologous competition
Homologous competition

130

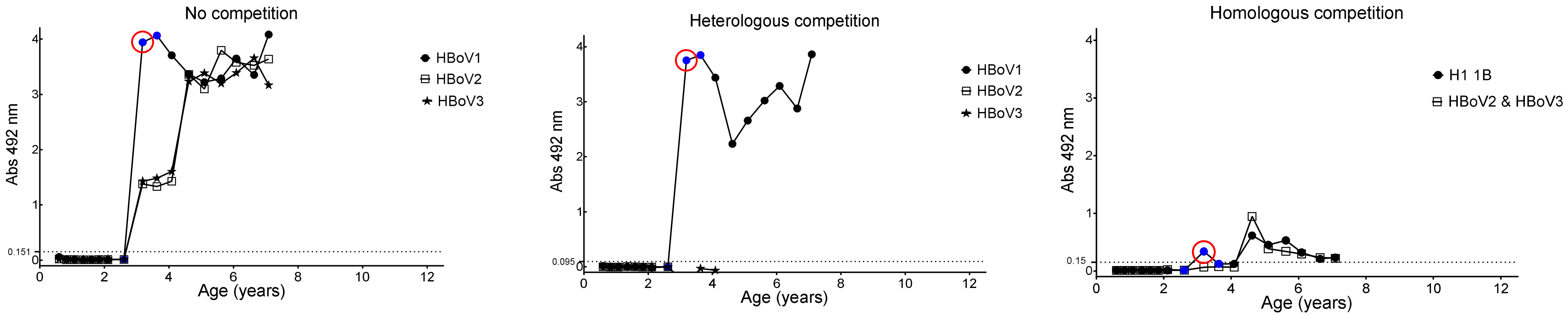
No competition
Heterologous competition
Homologous competition
HBoV1
HBoV2
HBoV3
H1 1B
HBoV2 & HBoV3
Abs 492 nm
Age (years)
0.151
0.095
0.15
0
1
2
3
4
6
8
10
12
